# Imatinib Reverses Doxorubicin Resistance by Affecting Activation of STAT3-Dependent NF-κB and HSP27/p38/AKT Pathways and by Inhibiting ABCB1

**DOI:** 10.1371/journal.pone.0055509

**Published:** 2013-01-31

**Authors:** Jonathan T. Sims, Sourik S. Ganguly, Holly Bennett, J. Woodrow Friend, Jessica Tepe, Rina Plattner

**Affiliations:** Department of Molecular and Biomedical Pharmacology, University of Kentucky School of Medicine, Lexington, Kentucky, United States of America; University of Kentucky, United States of America

## Abstract

Despite advances in cancer detection and prevention, a diagnosis of metastatic disease remains a death sentence due to the fact that many cancers are either resistant to chemotherapy (conventional or targeted) or develop resistance during treatment, and residual chemoresistant cells are highly metastatic. Metastatic cancer cells resist the effects of chemotherapeutic agents by upregulating drug transporters, which efflux the drugs, and by activating proliferation and survival signaling pathways. Previously, we found that c-Abl and Arg non-receptor tyrosine kinases are activated in breast cancer, melanoma, and glioblastoma cells, and promote cancer progression. In this report, we demonstrate that the c-Abl/Arg inhibitor, imatinib (imatinib mesylate, STI571, Gleevec), reverses intrinsic and acquired resistance to the anthracycline, doxorubicin, by inducing G2/M arrest and promoting apoptosis in cancer cells expressing highly active c-Abl and Arg. Significantly, imatinib prevents intrinsic resistance by promoting doxorubicin-mediated NF-κB/p65 nuclear localization and repression of NF-κB targets in a STAT3-dependent manner, and by preventing activation of a novel STAT3/HSP27/p38/Akt survival pathway. In contrast, imatinib prevents acquired resistance by inhibiting upregulation of the ABC drug transporter, ABCB1, directly inhibiting ABCB1 function, and abrogating survival signaling. Thus, imatinib inhibits multiple novel chemoresistance pathways, which indicates that it may be effective in reversing intrinsic and acquired resistance in cancers containing highly active c-Abl and Arg, a critical step in effectively treating metastatic disease. Furthermore, since imatinib converts a master survival regulator, NF-κB, from a pro-survival into a pro-apoptotic factor, our data suggest that NF-κB inhibitors may be ineffective in sensitizing tumors containing activated c-Abl/Arg to anthracyclines, and instead might antagonize anthracycline-induced apoptosis.

## Introduction

The goal of chemotherapy is to kill disseminated cancer cells and prevent metastatic progression; however, many cancers are intrinsically resistant to conventional chemotherapeutic agents, and others that initially respond, develop resistance (acquired) during treatment [1]. The anthracycline, doxorubicin, a topoisomerase II inhibitor, is used to treat many cancers, such as triple-negative (ER^−^, PR^−^, Her-2^−^) breast cancer; however, resistance arises for many cases [2], [3]. For other cancers, such as melanoma, doxorubicin is not routinely utilized due to intrinsic resistance [4]. Thus, although doxorubicin is a highly effective agent, its use is limited due to resistance as well as due to its narrow therapeutic window (e.g. cardiac toxicity) [5]. Drug resistance has been linked to upregulation of efflux molecules (ABC transporters), which play a role in both intrinsic and acquired chemoresistance [6]. Numerous transporters have been implicated in chemoresistance; however, ABCB1 (MDR-1, P-gp), ABCC1 (MRP1), and ABCG2 (BCRP) have been most extensively studied [7]. Activation of a variety of pathways including FOXO3a, PI3K/Akt, NF-κB, and extracellular signal-regulated kinase (ERK), as well as HSP27 depletion have been implicated in ABC transporter upregulation [8]–[10].

Activation of proliferation and survival signaling pathways also contribute to chemoresistance. Signal Transducer and Activator of Transcription (STAT3) and NF-κB transcription factors, promote oncogenesis, increasing proliferation, survival, invasion, and metastasis by promoting transcription of pro-proliferative, pro-invasive, and anti-apoptotic genes [11]–[14]. The NF-κB family, which consists of p65 (RelA), RelB, p50/105 (NF-κB1), c-Rel, and p52/p100 (NF-κB2), are constitutively activated in many cancers [14]. NF-κB is activated via the canonical pathway by Inhibitor of κB kinase (IKKβ)–dependent phosphorylation and degradation of IκB (which normally binds and inhibits p50/p65 dimers from entering the nucleus) [14]. NF-κB dimers translocate into the nucleus where they bind NF-κB response elements and promote transcription [14]. NF-κB post-translational modifications (e.g. phosphorylation and acetylation) regulate its nuclear localization, DNA binding, oligomerization, interaction with coactivators/corepressors, and transactivation [14]. NF-κB promotes survival by inducing expression of anti-apoptotic proteins; however, accumulating evidence suggests that NF-κB also can promote apoptosis and serves as a tumor suppressor in some tumor types [14], [15]. Similarly, in some cell types and in response to some agents, NF-κB promotes chemoresistance whereas in other cell types DNA damaging agents activate NF-κB and convert it into a repressor that inhibits transcription of anti-apoptotic genes and promotes apoptosis [16]–[22]. Activation of the phosphoinositide 3-kinase (PI3K/)Akt pathway also is critical for cancer development, progression and chemoresistance [23], [24]. PI3K activates PDK1, which membrane localizes and phosphorylates Akt (T308) [24]. Akt is further activated by phosphorylation on a second residue (S473) via mTORC2 (in response to growth factors), p38/MK2 (MAPKAPK-2; in neutrophils and neuronal cells) or DNA-PK (in response to insulin, pervanadate in glioblastoma cells) [25]–[28]. Active p38/MK2 promotes phosphorylation of the scaffold protein, HSP27, which recruits Akt, and Akt is phosphorylated on S473. Active Akt, in turn, phosphorylates HSP27, mediating its dissociation from the complex, and Akt also phosphorylates numerous other substrates involved in cell proliferation, survival, translation, and metabolism [24], [25], [28], [29].

The Abl family of non-receptor tyrosine kinases (c-Abl/Arg) are most known for their involvement in the development of human leukemia; however, recently, we provided evidence that they also promote solid tumor progression [30]–[33]. Here, we demonstrate that inhibition of c-Abl/Arg in cells with high c-Abl/Arg activity abrogates doxorubicin resistance by inducing G2/M cell cycle arrest and apoptosis, blocking activation of a novel Akt survival pathway, promoting repression of NF-κB targets, and preventing expression and function of ABCB1. Thus, in combination with c-Abl/Arg inhibitors, doxorubicin may be effective in cancers not previously treated with this agent (e.g. melanoma), and c-Abl/Arg inhibitors may decrease doxorubicin toxicity in cancers where the drug currently is used (e.g. breast cancer) by decreasing the doxorubicin dose required for effective treatment.

## Materials and Methods

### Cell Lines and Reagents

MDA-MB-435s cells are a spindle-shaped, highly metastatic variant of MDA-MB-435 cells obtained from ATCC (American Type Tissue Collection, Manassas, VA) [33]. DNA STR analysis confirmed that these cells are genetically identical to melanoma M14, and therefore, are referred to as 435s/M14 [33], [34]. Here, we created a drug-resistant variant of 435s/M14 (435s/M14-DR) via step-wise treatment with increasing concentrations of doxorubicin (using a maximum dose of 100 nM) [35]. 435s/M14 and 435s/M14-DR cells were cultured in DMEM/10% FBS (fetal bovine serum)+insulin (10 µg/ml). WM3248 melanoma cells (derived from a vertical growth phase (VGP) primary melanoma by Dr. Meenhard Herlyn, Wistar Institute, Philadephia, PA) [36] were cultured in MCDB153+L-15 (80∶20 mixture, respectively)/2% FBS, insulin (5 µg/ml), calcium chloride (1.68 mM). BT-549 breast cancer cells (obtained from Rolf Craven, University of Kentucky, Lexington KY who obtained the cells from ATCC) were cultured in DMEM/10% FBS. Genetic analysis showed 100% identity with ATCC BT-549 cells (Figure S1) [37]. MDA-MB-468 breast cancer cells (obtained from Eric Stanbridge, University of California, Irvine who obtained the cells from ATCC) were cultured in MEM/10% FBS, supplemented with sodium pyruvate (10 µg/ml). We expressed constitutively active STAT3 (STAT3C) stably in 435s/M14 cells (obtained from ATCC) [33]. MCF-7 cells (obtained from ATCC) were stably transfected with ABCC1 cDNA by Christian Paumi [38]. Cells expressing GFP-tagged PI3K (E545K) (Addgene; Cambridge, MA; donated by Jean Zhao) were obtained by transfection (Lipofectamine 2000; Invitrogen, Carlsbad, CA) followed by G418 (900 µg/ml)/puromycin (1 µg/ml) selection, and flow sorting GFP-positive cells (University of Kentucky Flow Cytometry Facility). The 3X-NF-κB reporter construct was provided by Dr. Denis Guttridge (Ohio State University; Columbus, OH) [39]. Migr1-c-Abl and pK1-Arg [40] were mutated to create imatinib-resistant c-Abl/Arg expression plasmids (mutation of threonine 315 to isoleucine; AblT/ArgT) [41] (GenScript; Piscataway, NJ). pK1-ArgT315I was transfected into cells, and expressing cells were obtained following puromycin selection. ArgT315I-expressing cells were transiently transfected with Migr1-AblT315I to create c-AblT315I/ArgT315I-expressing cells.

Imatinib (imatinib mesylate, Gleevec, STI571) and nilotinib were obtained from Novartis (Basal, Switzerland). Imatinib was dissolved in water (10 mM) and stored at −80°C, while nilotinib (10 mM) was dissolved in DMSO, and stored at 4°C. Doxorubicin, paclitaxel, camptothecin, 5-fluorouracil (5-FU), cisplatin, LY294002, and verapamil were purchased from Sigma (St. Louis, MO), and rhodamine 123 was purchased from Invitrogen (Carlsbad, CA). Silencer and Silencer select siRNAs were obtained from Applied Biosystems/Ambion (Carlsbad, CA): c-Abl (1336-20 nM), Arg (1478-20 nM), ABCB1 (s10418-5 nM), p65 (s11915-10 nM), and STAT3 (s743-1 nM). The following antibodies were purchased commercially: PARP (poly(ADP-ribose) polymerase; sc-8007), α-tubulin, p65, and Arg (Santa Cruz Biotechnologies; Santa Cruz, CA); GAPDH and c-Abl (8E9) (BD Biosciences; Chicago, IL); Lamin A/C, ABCB1, ABCG2, and ABCC1 (Milllipore; Temecula, CA); β-actin and FLAG (Sigma; St. Louis, MO); HSP27, XIAP, and cIAP1 (R&D Systems; Minneapolis, MN); and STAT3, phospho-STAT3 (Y705), phospho-Crk/CrkL (Y221/Y207), phospho-p38 (T180/T182), p38, Akt, phospho-p65 (S536), caspase-3, and phospho-Akt (S473) (Cell Signaling Technology; Danvers, MA).

### Cell Lysis/Western Blotting

Treated cells were lysed in RIPA buffer containing fresh phosphatase/protease inhibitors (50 mM Tris pH 7.5, 150 mM NaCl, 1% triton-X 100, 0.1% SDS, 1% sodium deoxycholate, 1 mM pefabloc, 10 µg/ml leupeptin, 10 µg/ml aprotinin, 1 mM sodium orthovanadate, 25 mM sodium fluoride), protein quantitated by Lowry DC (Bio-Rad; Hercules, CA), equal protein was loaded on SDS-PAGE gels, and gels transferred to nitrocellulose. Western blots were performed as described in the antibody manufacturers' protocols. For ABC transporter blots, SDS-PAGE sample buffer was added to lysates, lysates were frozen at −80°C, and thawed lysates were loaded on SDS-PAGE gels without boiling.

### CellTiter-Glo Viability Assay (Promega; Madison, WI) [42]

Cells were plated in 96-well plates in triplicate in 100 µl of medium, refreshed with media containing drugs the following day when cells were 30–40% confluent, and harvested 72 h later. CellTiter-Glo reagent (100 µl) was added to each well, the plates were rocked for 2′, incubated at room temperature for 10′, 100 µl was removed from each well, transferred to an opaque 96-well plate, and luminescence (total light emitted, 10″/well) measured with a Synergy 2 microplate reader (Biotek; Winooski, VT).

### Proliferation Assays

Tritiated thymidine assays [42]. Cells were plated in 24-well plates in triplicate, drug-treated the following day (30–40% confluent), and harvested after 72 h. Cells were pulsed with tritiated thymidine (2.5 µCi; 2 h), washed with PBS, incubated in 10% trichloroacetic acid, radioactivity was solubilized in 0.2N NaOH, and analyzed on a scintillation counter. Cell cycle analyses. Cells were plated in 100 mm dishes, refreshed with media containing drugs the following day when cells were 30–40% confluent, and harvested after 72 h. Cells were labeled with bromodeoxyuridine (BrdU, 10 µM) for 1 h at 37°C, trypsinized, washed and permeabilized with ethanol (70%), stained with fluorescein isothiocyanate (FITC)-conjugated anti-BrdU antibody and propidium iodide (PI; 5 µg/ml), and analyzed by fluorescence-activated cell sorting (FACS) using Cell Quest software (BD Biosciences) and Modfit analysis (Verity Software House, Topsham, ME).

### Apoptosis Assays

PARP and caspase-3 cleavage [42]. Cells were plated in 60 mm dishes, and treated with drugs the following day (30–40% confluent). After 40 h, attached and detached cells were lysed in RIPA buffer (see above), and blots were incubated with PARP, caspase-3 and GAPDH antibodies. Fluorescent Caspase-3/7 assay (Sigma; St. Louis, MO) [43]. Cells were plated in 6-well dishes in triplicate, drug-treated the following day when cells were 30–40% confluent, and attached and detached cells were lysed 40 h later. Lysate (5 µl) was incubated with substrate (200 µl, diluted 1∶3), and fluorescence detected at 360 nm/460 nm (excitation/emission) with a Synergy 2 microplate reader (Biotek; Winooski, VT). Annexin-V FACS analysis [32]. Cells were plated in 100 mm dishes, and treated with drugs the following day when cells were 30–40% confluent. After 40 h, cells were trypsinized, washed in DMEM (-phenol red, +Ca^2+^)/5% FBS, counted, and cells (1×10^5^) were incubated with Annexin-V-APC (5 µl) and propidium iodide (50 µg/ml) for 15′ prior to FACS analysis.

### Doxorubicin Accumulation Assays [44]

Subconfluent cells were incubated with either rhodamine 123 (0.5 µg/ml) or doxorubicin (10 µM) in the presence of verapamil (10 µM) or imatinib (40 µM) for 30′, washed extensively, incubated with verapamil or imatinib for an additional 45′, and analyzed by FACS to assess rhodamine 123 or doxorubicin intracellular retention.

### NF-κB Reporter Assays

Cells were transfected (Lipofectamine 2000, Invitrogen, Carlsbad, CA) with 3X-NF-κB luciferase reporter and pcDNA3.1 plasmids (10∶1 ratio), selected with G418 (900 µg/ml), and clones were pooled. Cells stably expressing the reporter were plated in triplicate, treated with doxorubicin, imatinib or the combination for 24 h, lysed in lysis buffer (Promega; Madison, WI), lysate was incubated with luciferin (Promega; Madison, WI) for 2″, and luminescence resulting from luciferase activity measured with the Synergy 2 microplate reader (total light emitted, 10″/well).

### Nuclear fractionation assays [42]

Cells were plated in 60 mm dishes, and nuclear fractions were isolated using the NE-PER kit (Pierce; Rockford, IL) as described in the manufacturer's protocol. Fraction purity was assessed by blotting with Lamin A/C (nuclear) and α-tubulin (cytoplasm) antibodies.

### Kinase Assays [45]

Assays were performed as described previously. Briefly, cells were serum-starved overnight, lysed in a Triton-X-100 based lysis buffer, c-Abl and Arg immunoprecipitated, washed with a series of stringent buffers, incubated in a kinase reaction with ^32^P-γ-ATP, 1 µM cold ATP and GST-Crk for 40′ at room temperature. Kinase reactions were run on SDS-PAGE gels, dried, exposed to film, and bands quantitated using a STORM phosphoimager and ImageQuant Software (Molecular Dynamics, GE Heathcare; Piscataway, NJK).

### Statistics

Statistical analyses were performed using Sigma Stat for Windows (Systat Software, Inc.; San Jose, CA) or the Vassar Website. Combination Index (CI) values were calculated with CalcuSyn software (Biosoft; Cambridge, UK) using single drug dose response curves and combination dose response curves using 3–4 doses of each drug. For simplicity, response curves shown in the figures demonstrate the effects of varying doxorubicin doses in combination with one imatinib dose. Student t-tests were used to analyze two sample comparisons; one-way ANOVA was utilized for multiple comparisons; and single sample t-tests were performed for comparisons against normalized controls. Two-tailed values are reported for all tests.

## Results

### Imatinib reverses doxorubicin resistance

**Figure 1:**
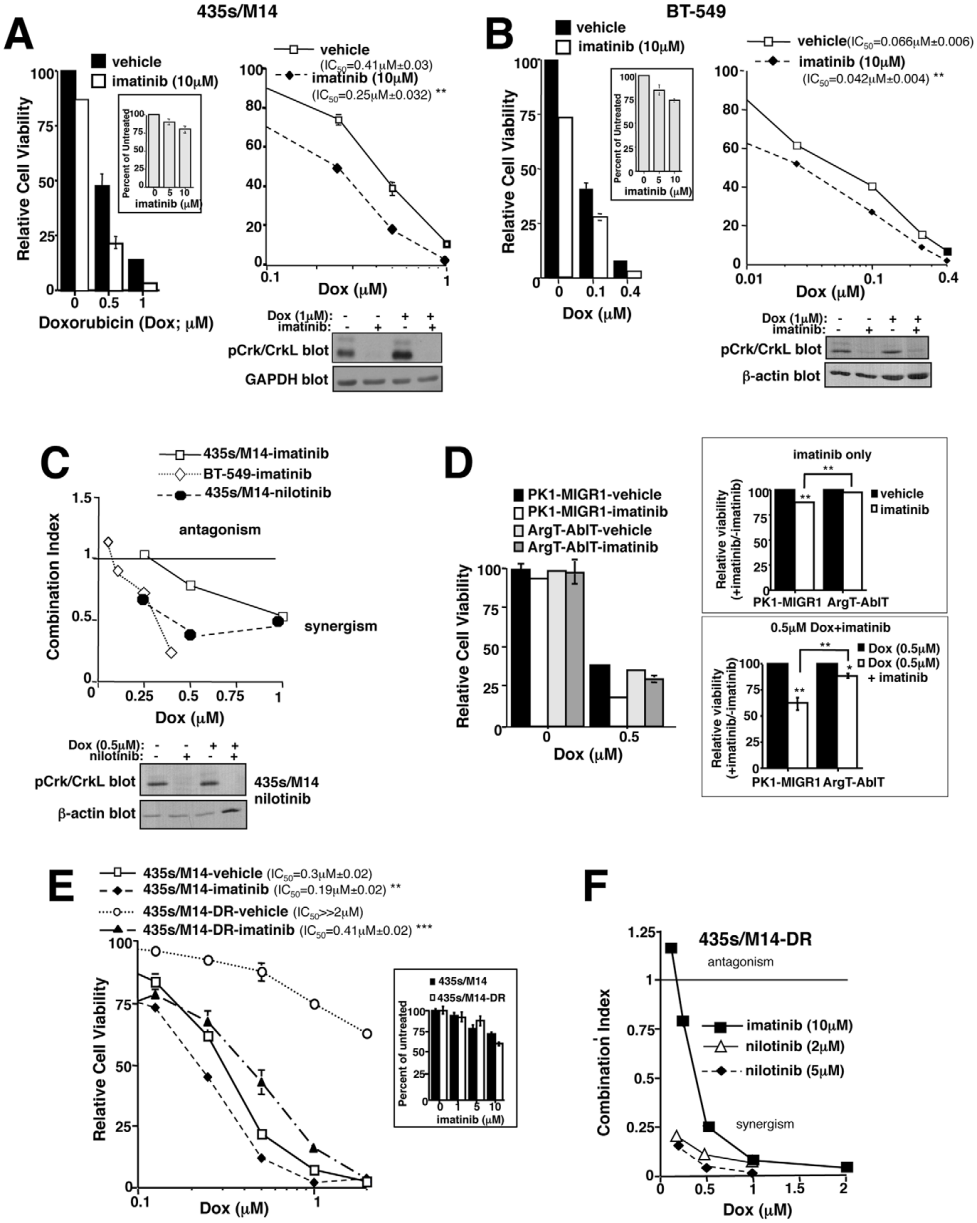
c-Abl/Arg inhibitors reverse doxorubicin resistance. (**A**) 435s/M14 melanoma and (**B**) BT-549 breast cancer cells were treated with doxorubicin/imatinib (72 h), and viability assessed by CellTiter-Glo. Mean±SEM for 3 independent experiments (left). Representative dose response curve (right). (**C,F**) Graphical representation of combination indices obtained with CalcuSyn software using dose response curves for each drug alone and in combination. >1-antagonism;  = 1-additive; <1-synergism. Graphs are representative of 3 independent experiments. (**D**) Cells stably expressing imatinib-resistant mutant Arg (ArgT) were transiently transfected with imatinib-resistant c-Abl (c-AblT), treated with doxorubicin/imatinib (48 h), and viability assessed. Representative experiment (left). Mean±SEM of 3 independent experiments: imatinib alone (right, top) and imatinib+doxorubicin (right, bottom). (**E,F**) Parental (E) and acquired doxorubicin-resistant (F) cells were drug-treated (72 h), and viability assessed. Experiments were performed ≥3 times, and representative dose response curves are shown. Mean±SEM for 3 independent experiments is shown in Figure S3B. For all subfigures, some error bars are too small to visualize. *p<0.05, **p<0.01, ***p<0.001 (see methods).

To determine whether c-Abl/Arg inhibition prevents resistance to doxorubicin, we treated cancer cells expressing highly activated forms of c-Abl/Arg (assessed by *in vitro* kinase assay and phosphorylation of substrates, Crk/CrkL) [31], [33], with the c-Abl/Arg inhibitors, imatinib or nilotinib, alone or in combination with doxorubicin, and measured cell viability using the CellTiter-Glo assay, which quantitates ATP, a measure of metabolically active cells [42], [43]. Imatinib alone had a modest effect on cell viability; however, imatinib sensitized cancer cells to doxorubicin, shifting the curves to the left and reducing the IC_50_s (Figure 1A,B and S2A,B). CalcuSyn software was utilized to calculate combination indices (CI), which indicate whether the effect of the two drugs together is greater than either alone using the dose response curves for each drug and the combination [42]. CI values less than one denote drug synergism, values equal to one signify additivity, and values greater than one indicate antagonism. Doxorubicin and imatinib synergistically inhibited the viability of 435s/M14 and WM3248 melanoma cells and BT-549 triple-negative (ER^−^, PR^−^, HER-2^−^) breast cancer cells, and inhibited the viability of MDA-MB-468 triple-negative breast cancer cells in an additive manner (Figure 1C and S2C). A dose of 10 µM imatinib was used for these studies because this physiologically relevant dose is required to effectively inhibit c-Abl/Arg kinase activities [31]. Moreover, nilotinib, a second generation inhibitor that is more specific for c-Abl/Arg [46], was highly synergistic with doxorubicin (Figure 1C and S2D). Low doses of doxorubicin had little effect on c-Abl/Arg activity (Figure 1B,C; assessed by measuring phosphorylation of endogenous substrates, Crk/CrkL [33]), whereas higher doses activated c-Abl/Arg (1 µM, Figure 1A). None of the cell lines examined express PDGFRα,β, or c-Kit, other imatinib/nilotinib targets, except MDA-MB-468 (c-Kit) [31], [33]. As expected, melanoma cells were intrinsically more resistant to doxorubicin than breast cancer cells (435s/M14, IC_50_ = 0.41 µM; WM3248, IC_50_ = 0.41 µM; BT-549, IC_50_ = 0.066 µM; MDA-MB-468, IC_50_ = 0.1 µM); however, imatinib sensitized both cell types to doxorubicin (Figure 1A,B and S2A,B). Doxorubicin is considered front-line therapy for “triple-negative” breast cancers (ER^−^, PR^−^, Her-2^−^; e.g. BT-549) [2]; however, doxorubicin is not used to treat melanoma due to intrinsic resistance. Here, we demonstrate that addition of nilotinib to a doxorubicin regimen can convert more resistant melanoma cells (IC_50_ = 0.41 µM) into cells that have a similar doxorubicin sensitivity as MDA-MB-468 breast cancer cells (435s/M14-nilotinib+doxorubicin. IC_50_ = 0.16 µM vs. MDA-MB-468-doxorubicin, IC_50_ = 0.1 µM; Figure S2B,D).

Next, we tested whether c-Abl/Arg are the targets of imatinib during doxorubicin-mediated sensitization. Unfortunately, transfection of cells with c-Abl/Arg-specific siRNAs reduced cell proliferation [32], [33], decreasing the effectiveness of doxorubicin, which targets proliferating cells. Furthermore, it was not possible to transfect siRNAs after doxorubicin treatment, as this resulted in inefficient knockdown (data not shown). Moreover, cells stably expressing c-Abl/Arg shRNAs could not be obtained, likely due to the requirement of c-Abl/Arg for cell growth (data not shown). Therefore, we utilized an alternative strategy which involved expressing imatinib-resistant mutant forms of c-Abl and Arg (c-AblT315I, ArgT315I, abbreviated AblT/ArgT), and determining whether their expression rescues imatinib-mediated chemosensitization. Expression of the mutant forms prevented imatinib from inhibiting c-Abl/Arg activity (assessed by blotting with an antibody to the c-Abl/Arg phosphorylation sites on substrates, Crk and CrkL; Figure S2E). Significantly, expression of imatinib-resistant forms of c-Abl and Arg prevented imatinib-mediated sensitization to doxorubicin (Figure 1D), whereas expression of either c-AblT315I or ArgT315I alone only partially abrogated imatinib-mediated sensitization (data not shown). These data indicate that imatinib-mediated reversal of doxorubicin resistance is due, in large part, to inhibition of c-Abl and Arg.

### Cells that acquire high-level doxorubicin resistance are extremely sensitive to imatinib/nilotinib in the presence of doxorubicin

Although chemotherapeutic agents sometimes kill the majority of cells, residual, highly resistant cells often remain, which are very aggressive and metastatic. In order to mimic outgrowth of highly resistant, metastatic cells following treatment with chemotherapeutic agents, we cultured 435s/M14 melanoma cells with increasing concentrations of doxorubicin over the course of 8 months (435s/M14-DR). 435s/M14-DR cells were highly resistant to doxorubicin (1 µM reduced viability by 15% as compared to 80% in parental cells; IC_50_≫2 µM), and continued to express highly active c-Abl/Arg (Figure 1E and S3A,B). Significantly, imatinib and nilotinib dramatically sensitized highly resistant cells to doxorubicin (Figure 1E,F and S3B,C). These data indicate that c-Abl/Arg inhibitors not only are involved in reversing intrinsic doxorubicin resistance, but also abrogate acquired resistance.

### Imatinib reverses doxorubicin resistance by preventing G2/M arrest and inhibiting apoptosis

**Figure 2:**
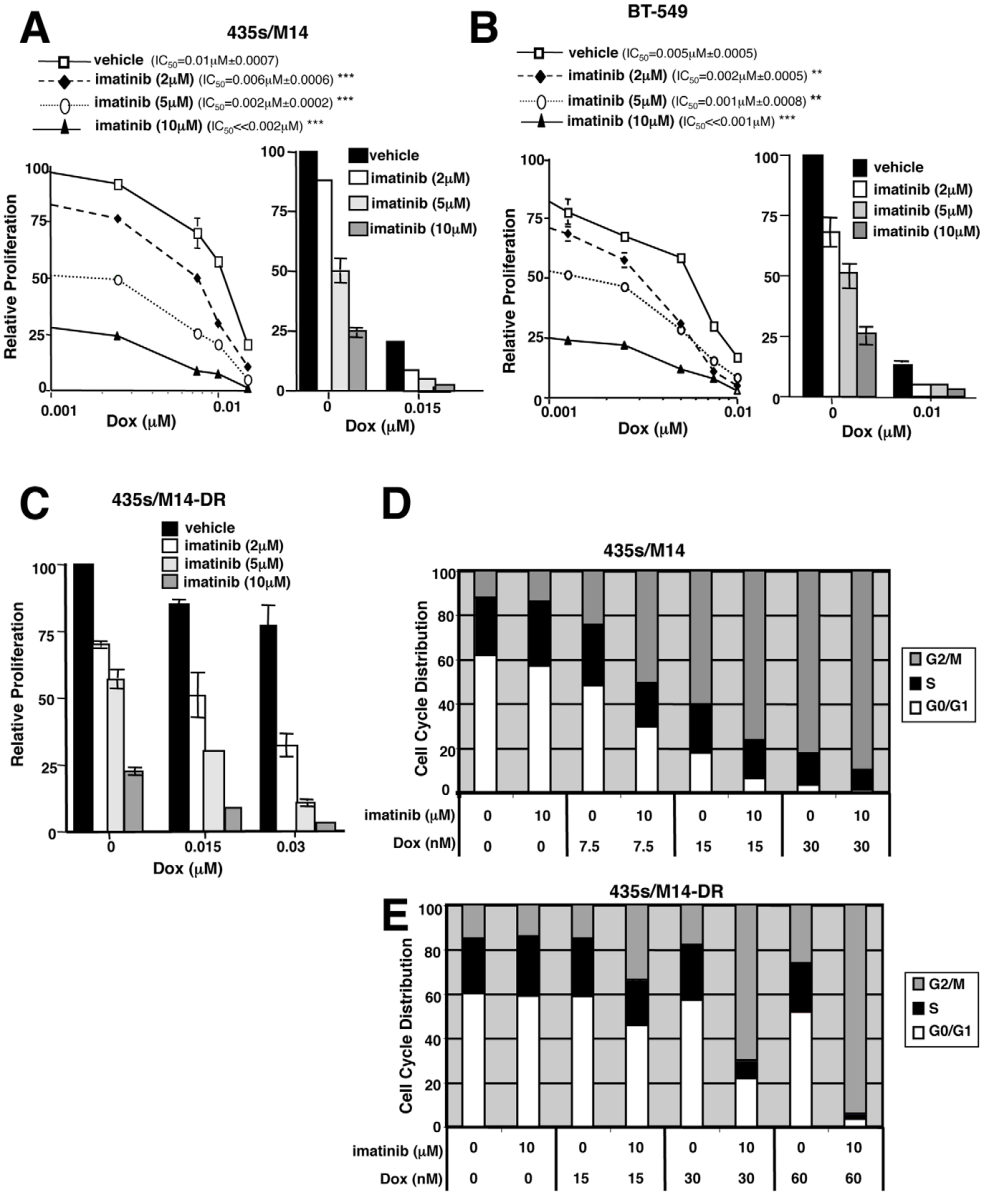
Imatinib abrogates doxorubicin resistance, in part, by potentiating doxorubicin-mediated G2/M arrest. (**A–C**) Proliferation was assessed in treated cells (72 h) by tritiated thymidine assay. Representative dose response curves (**A, B, left**) and Mean±SEM for 3 independent experiments (**A–C, right**). Some error bars are too small to visualize. (**C**) CI values: Dox(0.015 µM)+imatinib(2 µM) = 0.9±0.14; Dox(0.015 µM)+imatinib(5 µM) = 0.67±0.07; Dox(0.015 µM)+imatinib(10 µM) = 0.5±0.06; Dox(0.03 µM)+imatinib(2 µM) = 0.8±0.11; Dox(0.03 µM)+imatinib(5 µM) = 0.5±0.13; Dox(0.03 µM)+imatinib(10 µM) = 0.36±0.18. (**D, E**) Asynchronous drug-treated cells (72 h) were analyzed by FACS. Experiments were performed ≥3 times, and representative cell cycle distributions are shown. Mean±SEM for 3 independent experiments is shown in Figure S4E. **p≤0.01, ***p<0.001 (see methods).

Since viability is a balance of proliferation and apoptosis, we tested whether imatinib prevents chemoresistance by potentiating the anti-proliferative and/or pro-apoptotic effects of doxorubicin. Using tritiated–thymidine assays to assess cell proliferation, we show that imatinib alone inhibited proliferation of cells with intrinsic and acquired resistance, and addition of low doses of doxorubicin completely blocked cell proliferation (Figure 2A–C and S4A–D). To determine the mechanism by which imatinib synergizes with doxorubicin to prevent cell proliferation, BrdU/PI cell cycle analyses were performed on treated, asynchronously growing cells. Low dose doxorubicin treatment of parental cells resulted in a dose-dependent accumulation of cells in G2/M, and imatinib treatment dramatically potentiated the G2/M arrest (Figure 2D and S4E-left). In cells that acquired high-level doxorubicin resistance, doxorubicin alone had little effect on the cell cycle; however, addition of imatinib induced a dramatic blockade of cells in G2/M, using extremely low doxorubicin doses (30–60 nM; Figure 2E and S4E-right), indicating that imatinib reverses doxorubicin resistance, in part, by enhancing doxorubicin-mediated G2/M arrest.

**Figure 3:**
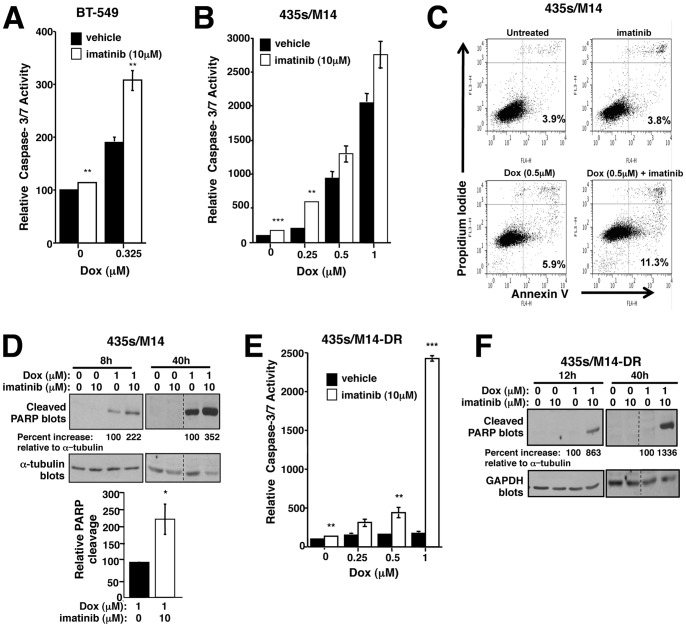
Imatinib reverses doxorubicin resistance by inhibiting cell survival. Caspase-3/7 activity (**A,B,E**), Annexin V staining (**C**), or PARP cleavage (**D,F**) were assessed in lysates from attached and detached treated cells (40 h unless otherwise indicated). Mean±SEM of 3 experiments (**A,B,E**) or representative of 3 independent experiments (**C,D,F**). Some error bars (A,B,E) are too small to visualize. *p<0.05, **p≤0.01, ***p<0.001 (see methods).

To examine whether imatinib abrogates chemoresistance by potentiating doxorubicin-mediated apoptosis, we assessed caspase-3/7 activity, PARP cleavage, and/or Annexin V staining in cells treated with higher doses of doxorubicin alone or in combination with imatinib. Imatinib alone modestly, but significantly, induced caspase-3/7 activity or PARP cleavage in all cell lines tested (Figure 3A,B and S4F). Significantly, imatinib potentiated doxorubicin-induced caspase-3/7 activity, PARP cleavage, and/or Annexin V staining in 435s/M14, BT-549 and WM3248 cell lines, but not in MDA-MB-468 (Figure 3A–D and S4F,G; data not shown). These data indicate that imatinib prevents intrinsic doxorubicin resistance in 435s/M14, BT-549, and WM3248 cells by inducing cell cycle arrest (at low doxorubicin doses) and abrogating survival (at higher doses). Conversely, in MDA-MB-468 cells, imatinib only inhibited proliferation and did not potentiate apoptosis, which explains why the effects of imatinib on viability were additive rather than synergistic. Interestingly, in cells that acquired high-level doxorubicin resistance, doxorubicin alone did not induce apoptosis; however, the addition of imatinib dramatically activated caspase-3/7 and induced PARP cleavage (Figure 3E,F). In conclusion, imatinib reverses both intrinsic and acquired resistance to doxorubicin by potentiating doxorubicin-mediated G2/M arrest and apoptosis.

### c-Abl contributes to upregulation of ABCB1, and ABCB1 overexpression promotes acquired doxorubicin resistance

**Figure 4:**
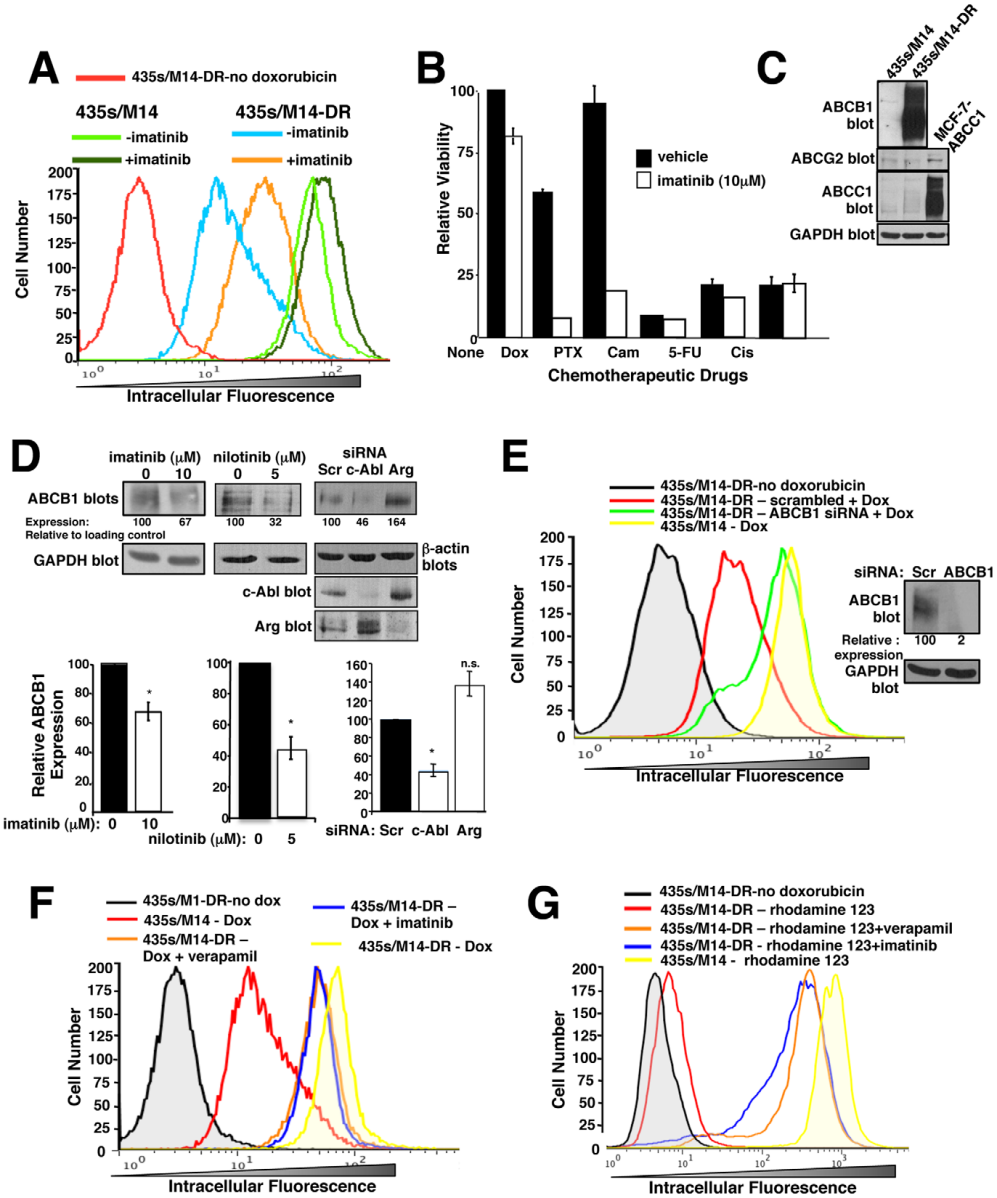
c-Abl inhibition decreases ABCB1 expression and activity. (**A**) Cells were treated with imatinib (72 h), washed, treated with doxorubicin (30′) in the absence of imatinib, and intracellular doxorubicin fluorescence assessed by FACS. The experiment was performed ≥3 times; a representative experiment is shown. (**B**) Cells were drug- treated (72 h), and viability was assessed. Graph is representative of 3 independent experiments. Some error bars are too small to visualize. PTX = paclitaxel (30 nM), Cam = camptothecin (1 µM), Cis = cisplatin (20 µM), 5-FU = 5-fluorouracil (500 µM). (**C,D**) Western blot analysis. Lysate from MCF-7 cells transfected with ABCC1 served as a positive control for ABCC1 expression. Blots are representative of ≥3 independent experiments. (**D**) Cells were imatinib or nilotinib-treated (48 h, left) or transfected with siRNAs (right), and lysates blotted with the indicated antibodies. Mean±SEM from 3 independent experiments (below). (**E**) siRNA-transfected cells were treated with doxorubicin (30′), washed, and intracellular doxorubicin fluorescence assessed. Graph is representative of 3 independent experiments. Representative Western blot (right). (**F, G**) Cells were treated with either doxorubicin (**F**) or rhodamine 123 (**G**) in the presence of the ABCB1 inhibitor, verapamil, or imatinib for 30′, washed, and incubated with verapamil or imatinib for an additional 45′, and rhodamine 123 or doxorubicin intracellular fluorescence assessed by FACS. Graphs are representative of 3 independent experiments. *p<0.05, **p≤0.01, ***p<0.001 (see methods). n.s. = not significant.

Chemoresistance can result from activation of cell proliferation/survival pathways and/or can be mediated by overexpression of multi-drug resistance (ABC) transporters, which efflux the chemotherapeutic agents [7]. To determine whether imatinib prevents doxorubicin intracellular accumulation, cells were treated with vehicle/imatinib for 72 h, washed, incubated with doxorubicin for 30′ in the absence of imatinib, and intracellular doxorubicin assessed in living cells. Doxorubicin possesses intrinsic fluorescence, which allows for its detection by flow cytometry. Less intracellular doxorubicin was observed in 435s/M14-DR cells as compared to parental cells (Figure 4A). Moreover, intracellular doxorubicin levels in parental cells were only slightly affected by treatment with imatinib, whereas in 435s/M14-DR cells, much more doxorubicin was retained in the cells following imatinib treatment, as evidenced by the curve shifting to the right (Figure 4A). These data indicate that imatinib-mediated chemosensitization likely occurs independent of an ABC transporter in parental cells, whereas in cells that acquire high-level resistance, chemosensitization likely involves inhibition of ABC transporter function. In order to identify the transporter involved in doxorubicin efflux in 435s/M14-DR cells, we tested whether the cells also are resistant to other chemotherapeutic agents from other chemotherapeutic classes. Interestingly, 435s/M14-DR cells were highly resistant to paclitaxel, and this resistance was abrogated by imatinib treatment (Figure 4B). However, 435s/M14-DR cells remained sensitive to camptothecin, 5-fluorouracil, and cisplatin (Figure 4B). Candidate transporters that efflux doxorubicin and paclitaxel include ABCB1, ABCG2, and ABCC1 [7]. Interestingly, 435s/M14-DR cells expressed dramatically elevated levels of ABCB1 protein in contrast to parental cells, which did not express ABCB1, whereas ABCC1 and ABCG2 were expressed at low levels in both cell lines (Figure 4C). Treatment of 435s/M14-DR cells with imatinib or nilotinib or transfection of cells with c-Abl but not Arg siRNA, partially inhibited ABCB1 expression (Figure 4D), indicating that c-Abl contributes to ABCB1 upregulation following acquired resistance to doxorubicin. Since prior imatinib treatment prevented doxorubicin from being effluxed from 435s/M14-DR cells (Figure 4A) even though imatinib was not present during the assay (i.e., during incubation with doxorubicin), and imatinib binding to ABC transporters is known to be a reversible process [47], these data indicate that imatinib increases intracellular doxorubicin retention in 435s/M14-DR cells, in part, by decreasing ABCB1 expression.

### Imatinib sensitizes cells that acquire high-level doxorubicin resistance to doxorubicin, in part, by inhibiting ABCB1 function

**Figure 5:**
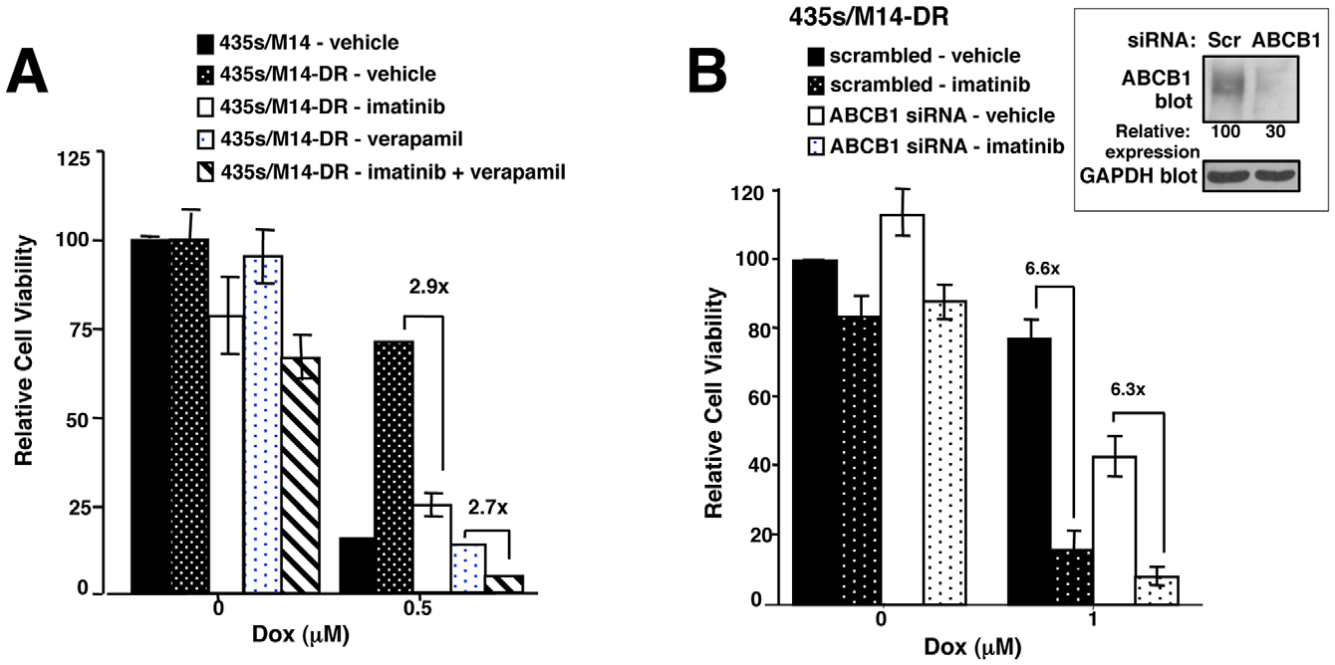
Resistance to doxorubicin occurs via ABCB1-dependent and ABCB1-independent mechanisms. (**A, B**) ABCB1 was inhibited with verapamil (**A**) or silenced with siRNA (**B**), cells were treated with imatinib (10 µM) and/or doxorubicin (0.5 µM) for 72 h, and cell viability assessed. Mean±SEM from 3 independent experiments. (A) Dox+imatinib, CI = 0.66±0.19; Dox+verapamil, CI = 0.14±0.04; Dox+imatinib+verapamil, CI = 0.47±0.5. Representative Western blot (right). Some error bars are too small to visualize.

Imatinib has been shown to be a substrate and/or inhibitor of ABCB1 and ABCG2 in leukemic cells [47]–[51]. Therefore, imatinib also may sensitize highly resistant cells to doxorubicin by directly inhibiting drug efflux. To confirm that ABCB1 mediates doxorubicin efflux and to ascertain whether imatinib specifically interferes with ABCB1-mediated efflux of doxorubicin, we performed doxorubicin accumulation assays in the absence or presence of imatinib, ABCB1 siRNA, or verapamil (ABCB1 inhibitor), and measured doxorubicin intracellular fluorescence. Silencing ABCB1 increased doxorubicin retention (Figure 4E), and imatinib promoted doxorubicin and rhodamine 123 (a ABCB1 substrate) accumulation, to a similar extent as verapamil (ABCB1 inhibitor; Figure 4F,G). Taken together, these studies demonstrate that imatinib directly inhibits ABCB1-mediated doxorubicin efflux in cells that acquire high-level doxorubicin resistance, in addition to preventing ABCB1 upregulation. Next, we assessed the functional impact of ABCB1 expression in cells that acquired doxorubicin resistance by assessing the effect of silencing/inhibiting ABCB1 on cell viability. Silencing ABCB1 or verapamil treatment dramatically sensitized resistant cells to doxorubicin (Figure 5A,B). However, imatinib further decreased the viability of cells treated with verapamil/doxorubicin or ABCB1 siRNA/doxorubicin (Figure 5A,B), indicating that inhibition of ABCB1 is not the only mechanism by which imatinib sensitizes acquired resistant cells to doxorubicin. In addition, unlike rhodamine 123, in the absence of transporter inhibitors, doxorubicin is not completely effluxed from 435s/M14-DR cells (Figure 4G vs. Figure 4F). Therefore, since 435s/M14-DR cells continue to retain some doxorubicin, but are resistant to its effects, this indicates that transporter-independent pathways also contribute to acquired doxorubicin resistance.

### Imatinib potentiates doxorubicin-induced apoptosis by inhibiting STAT3-dependent survival pathways

**Figure 6:**
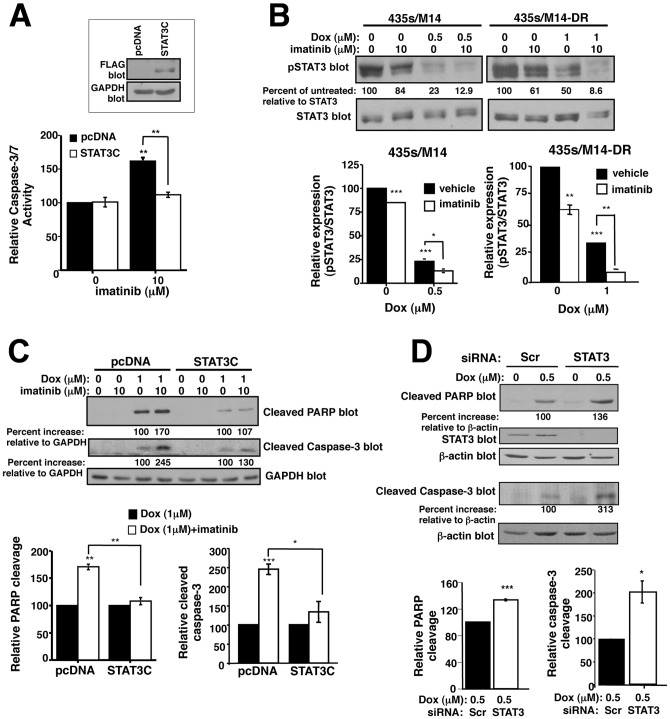
Imatinib promotes apoptosis in response to doxorubicin by inhibiting STAT3-dependent survival pathways. (**A**) 435s/M14 cells, stably transfected with pcDNA or STAT3C, were treated with imatinib (40 h), and caspase 3/7 activity assessed in lysate from attached/detached cells. Graph is Mean±SEM from 3 independent experiments. Representative Western blot (above). (**B**) Parental (435s/M14) and acquired doxorubicin-resistant (435s/M14-DR) cells were drug-treated (40 h), and lysates from attached/detached cells were blotted with antibodies. Graphs are Mean±SEM from 3 independent experiments. (**C**) Western blots of lysates from 435s/M14 cells expressing pcDNA or STAT3C, treated with imatinib/doxorubicin (40 h). Graphs are Mean±SEM from 3 independent experiments. (**D**) Lysates from drug-treated (40 h), attached/detatched 435s/M14 cells transfected with STAT3 siRNA were blotted. Graphs are Mean±SEM from 3 independent experiments. For all subfigures, some error bars are too small to visualize. *p<0.05, **p≤0.01, ***p<0.001 (see methods).

Since ABC transporters are not involved in intrinsic resistance, and transporter-independent pathways contribute to acquired resistance, we set out to identify signaling pathways by which imatinib prevents survival during doxorubicin treatment. Previously, we showed that c-Abl/Arg promote phosphorylation of the STAT3 transcription factor (Y705) in a variety of cancer cell lines [32], [33]. STAT3 drives cancer cell proliferation, survival, invasion, and metastasis, and also has been implicated in chemoresistance; thus, c-Abl/Arg may drive doxorubicin resistance by activating STAT3 [12], [32], [52]. In the absence of doxorubicin, stable expression of a constitutively active form of STAT3 (STAT3C) prevented the modest imatinib-mediated activation of caspase-3/7, indicating that imatinib prevents cancer cell survival by inhibiting activation of STAT3 (Figure 6A). Next, we tested whether STAT3 dephosphorylation is required for imatinib to reverse doxorubicin resistance. Doxorubicin inhibited STAT3 phosphorylation in parental cells, which was potentiated by imatinib (Figure 6B-left). Interestingly, doxorubicin also inhibited STAT3 phosphorylation in cells that acquired doxorubicin resistance (albeit to a lower extent; Figure 6B-right) even though doxorubicin is efficiently effluxed by ABCB1 in these cells (Figure 4). Expression of STAT3C partially prevented imatinib from potentiating doxorubicin-mediated inhibition of viability, proliferation, and cell cycle progression (Figure S5), and completely blocked the ability of imatinib to cooperate with doxorubicin to induce PARP and caspase-3 cleavage (Figure 6C). Furthermore, silencing STAT3 potentiated doxorubicin-induced PARP and caspase-3 cleavage similar to the effects observed with imatinib (Figure 6D). Taken together, these data indicate that doxorubicin-mediated inhibition of STAT3 phosphorylation is required for doxorubicin to kill cancer cells, and imatinib reverses doxorubicin resistance by preventing STAT3 phosphorylation.

### Imatinib promotes doxorubicin-induced NF-κB-mediated repression of anti-apoptotic genes

**Figure 7:**
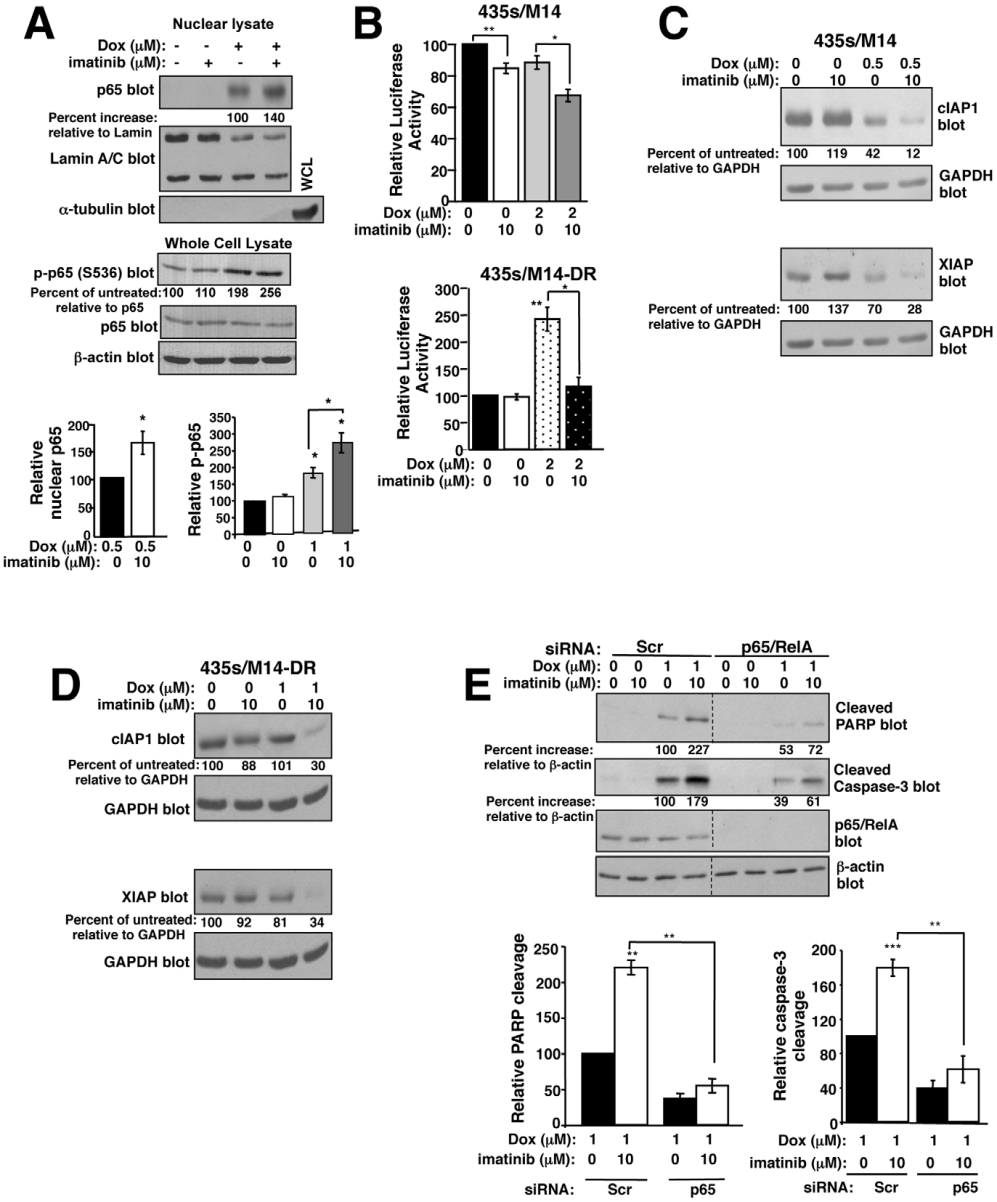
Imatinib abrogates doxorubicin resistance, in part, by increasing p65 nuclear translocation, and inhibiting expression of NF-κB targets. (**A**) Nuclear lysates from doxorubin (0.5 µM-top; 1 µM-bottom)/imatinib (10 µM)-treated 435s/M14 cells (8 h) were blotted. Graphs are Mean±SEM from 3 independent experiments. (**B**) Parental (435s/M14) and doxorubicin-resistant (435s/M14-DR) cells, stably expressing a 3X-NF-κB-luciferase reporter, were drug treated (8 h), and luciferase activity assessed. Graphs are Mean±SEM from 3 independent experiments. (**C, D**) Lysates from treated cells were analyzed by Western blot (40 h treatment). Representative blots are shown. Mean±SEM from 3 independent experiments is shown in Figure S6D,E. (**E**) 435s/M14 cells transfected with p65 siRNA were drug-treated (8 h), and PARP and caspase-3 cleavage assessed. Graphs are Mean±SEM from 3 independent experiments. *p<0.05, **p≤0.01 (see methods).

NF-κB promotes oncogenesis, increasing proliferation, survival, invasion, and metastasis by promoting the transcription of pro-proliferative, pro-invasive, and anti-apoptotic genes, and STAT3 promotes NF-κB transcriptional activity [14], [53]. Since c-Abl/Arg activate STAT3, we investigated whether c-Abl/Arg regulate NF-κB signaling. In the absence of doxorubicin, silencing or inhibiting c-Abl or Arg inhibited p65 nuclear localization, and decreased basal and TNF-α-induced NF-κB transcriptional activity (Figure S6A–C), indicating that c-Abl/Arg activate NF-κB signaling in cancer cells. To determine whether imatinib prevents survival in response to doxorubicin treatment by affecting NF-κB signaling, we assessed p65 nuclear localization and phosphorylation following imatinib/doxorubicin treatment. p65 phosphorylation regulates its acetylation and nuclear localization/retention [53], [54]. Surprisingly, in parental cells, doxorubicin treatment increased p65 (S536) phosphorylation and dramatically induced its nuclear localization, which was potentiated by imatinib, and doxorubicin and imatinib cooperated to decrease NF-κB transcriptional activity (Figure 7A,B-top). Therefore, NF-κB nuclear localization induced by doxorubicin correlated with decreased transcriptional activity, which is consistent with doxorubicin converting NF-κB into a transcriptional repressor [16], [19]. The modest effects we observed on transcriptional activity are in the same range as those previously reported [16]. Furthermore, imatinib enhanced NF-κB repressive activity, indicating that it acts to potentiate doxorubicin-mediated conversion of NF-κB into a transcriptional repressor. In contrast, in cells that acquired high-level doxorubicin resistance, doxorubicin increased NF-κB transcriptional activity, which was abrogated by imatinib (Figure 7B-bottom). Thus, in these cells, doxorubicin does not convert NF-κB into a repressor but instead promotes NF-κB transcriptional activity, and imatinib inhibits doxorubicin-mediated NF-κB activation. These data are significant as they indicate that NF-κB-mediated signaling mechanisms underlying doxorubicin resistance are not identical for cells with intrinsic vs. acquired resistance.

To confirm that NF-κB indeed acts as a repressor following doxorubicin+imatinib treatment in parental cells, we examined expression of NF-κB targets, such as those involved in inhibiting apoptosis (cIAP, XIAP). Many cancers overexpress cIAP1 and XIAP, and are addicted to their expression [15]. In parental cells, doxorubicin inhibited cIAP1/XIAP expression, and imatinib potentiated this inhibition (Figure 7C and S6D). In contrast, in cells that acquired high-level resistance, doxorubicin treatment had little effect on cIAP or XIAP expression (Figure 7D and S6E); however, addition of imatinib dramatically reduced cIAP1/XIAP expression (Figure 7D and S5E). These data are significant because they demonstrate that imatinib not only prevents NF-κB activation following doxorubicin treatment in cells that acquired doxorubicin resistance, but also converts NF-κB into a repressor that inhibits expression of cIAP1/XIAP. Significantly, silencing p65, in parental cells, reduced doxorubicin-mediated PARP and caspase-3 cleavage, and partially inhibited the potentiation induced by imatinib treatment (Figure 7E and S6F), which indicates that imatinib reverses doxorubicin resistance, in part, by inducing p65 nuclear translocation.

### Imatinib potentiates doxorubicin-mediated NF-κB nuclear localization and inhibition of NF-κB target expression by inhibiting activation of STAT3

**Figure 8:**
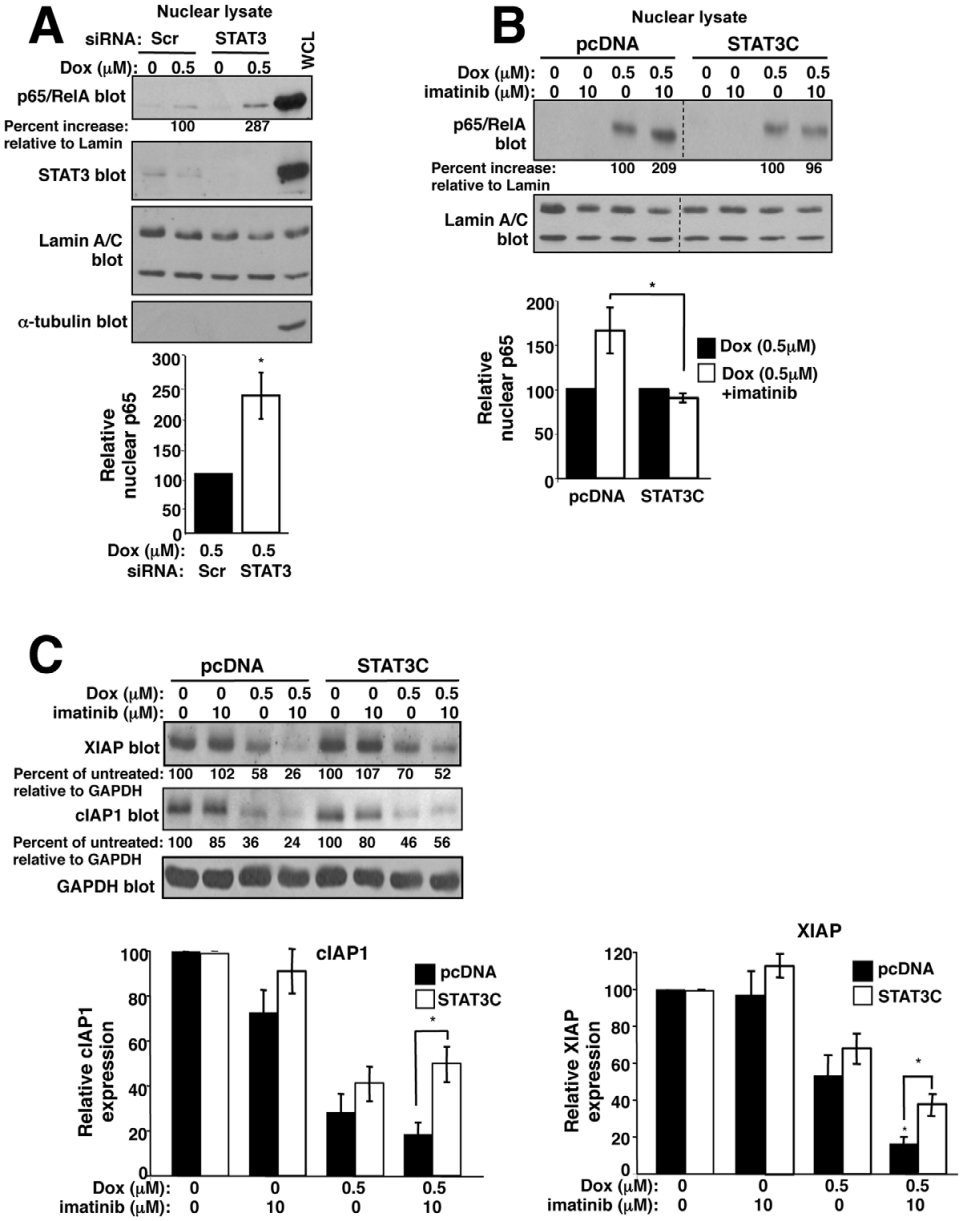
Imatinib promotes p65 nuclear translocation and inhibits NF-κB target expression in a STAT3-dependent manner. (**A**) 435s/M14 cells, transfected with STAT3 siRNA, were drug treated (8 h), and nuclear fractions analyzed by Western blot. Graph is Mean±SEM from 3 independent experiments. (**B**) 435s/M14 cells stably expressing pcDNA or STAT3C were drug treated (8 h), and nuclear fractions analyzed by Western blot. Graph is Mean±SEM of 3 independent experiments. (**C**) Lysates from drug-treated pcDNA and STAT3C-expressing cells (40 h), were analyzed by Western blot. Graphs are Mean±SEM from 3 independent experiments. For all subfigures, some error bars are too small to visualize. *p<0.05, **p≤0.01 (see methods).

Since STAT3 and NF-κB bind and cooperate to regulate transcription [52], c-Abl/Arg activate STAT3, and constitutive STAT3 activation prevents imatinib from reversing doxorubicin resistance (Figure 6C), we tested whether imatinib induces NF-κB-mediated apoptosis by inhibiting STAT3-dependent pathways. Significantly, silencing STAT3 potentiated doxorubicin-induced p65 nuclear localization, similar to imatinib (Figure 8A), and STAT3C expression prevented the imatinib-mediated increase in nuclear p65 (Figure 8B). Moreover, expression of STAT3C partially prevented imatinib from potentiating doxorubicin-mediated inhibition of cIAP1/XIAP expression (Figure 8C). Taken together, these data indicate that imatinib promotes p65 nuclear localization and inhibits NF-κB target expression by at least, in part, by inhibiting STAT3 activation.

### Imatinib abrogates doxorubicin resistance, in part, by preventing activation of a STAT3-dependent HSP27/p38/Akt pathway

**Figure 9:**
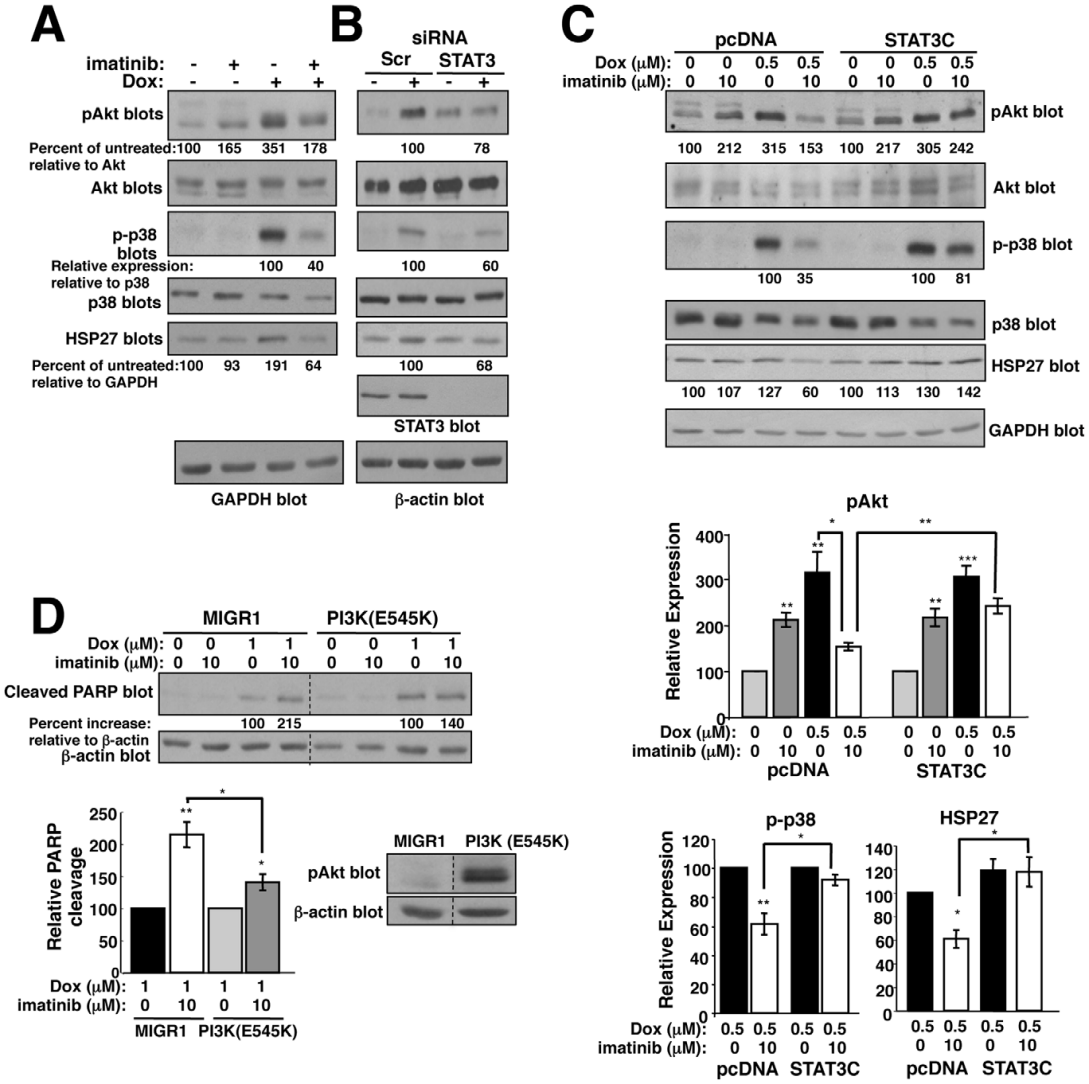
Imatinib abrogates doxorubicin resistance, in part, by preventing activation of a STAT3/HSP27/p38/Akt survival pathway. (**A, B**) 435s/M14 cells were treated with imatinib (10 µM) (**A**) or transfected with STAT3 siRNA (**B**), treated with doxorubicin (p38, Akt, 0.5 µM; HSP27, 1 µM) for 40 h, and lysate from attached/detached cells blotted with antibodies. Experiments were performed 3 times; representative blots are shown. Mean±SEM for 3 experiments is shown in Figure S7A. (**C**) 435s/M14 cells expressing pcDNA or STAT3C were drug treated (40 h), and lysate from attached/detached cells were blotted with antibodies. Graphs are Mean±SEM from 3 independent experiments (bottom). (**D**) 435s/M14 cells expressing vector or constitutively active PI3K (E545K) were drug-treated (8 h) and PARP cleavage assessed. Graph is Mean±SEM from 3 independent experiments. Representative Western blot (right). For all subfigures, some error bars are too small to visualize. *p<0.05, **p≤0.01, ***p<0.001 (see methods).

**Figure 10:**
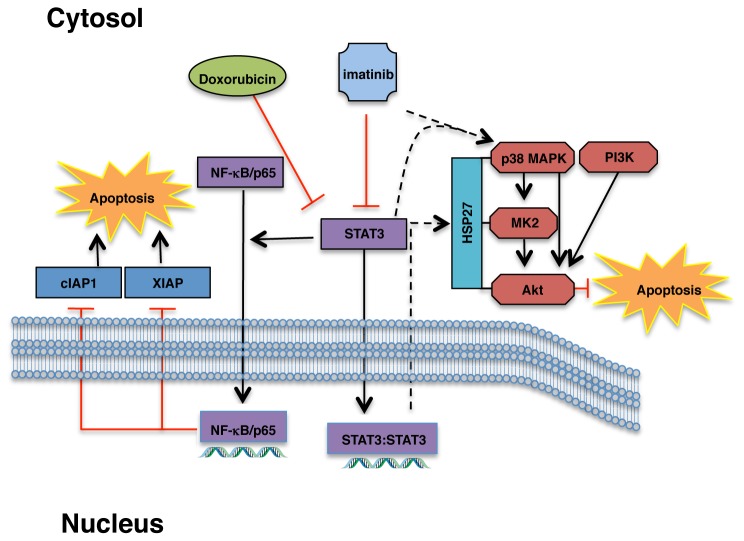
Mechanisms by which imatinib abrogates intrinsic doxorubicin resistance. Imatinib prevents activation of STAT3, which promotes doxorubicin-mediated NF-κB phosphorylation and nuclear translocation and NF-κB-mediated inhibition of expression of anti-apoptotic proteins such as cIAP1/XIAP. Imatinib-mediated inhibition of STAT3 also prevents activation of a HSP27/p38/Akt pro-survival pathway.

Expression of constitutively active STAT3 (STAT3C) completely prevented imatinib from increasing apoptosis following doxorubicin treatment (Figure 6C); however, silencing p65 only partially prevented imatinib from increasing doxorubicin-induced apoptosis (Figure S6F). These data indicate that imatinib reverses doxorubicin resistance via more than one STAT3-dependent pathway. PI3K/Akt are major mediators of cancer cell survival, and play a role in chemoresistance [23], [24]. Doxorubicin induced Akt phosphorylation in parental and highly resistant cells, and this was inhibited by addition of imatinib (Figure 9A and S7A,B). In neuronal cells and neutrophils, activation of a HSP27/p38/MK2 pathway mediates S473 phosphorylation following DNA damage/cell stress [26], [28], [29]. To test whether doxorubicin activates Akt in melanoma cells via a HSP27/p38 pathway, we examined p38 phosphorylation and HSP27 expression in doxorubicin/imatinib-treated cells. Indeed, doxorubicin induced expression of HSP27 and phosphorylation of p38, and imatinib dramatically inhibited HSP27/p-p38 induction (Figure 9A and S7A). Similar to imatinib, silencing STAT3 reduced Akt and p38 phosphorylation and HSP27 expression (Figure 9B, and S7C). Furthermore, expression of STAT3C prevented imatinib from reducing HSP27, phospho-p38, and phospho-Akt expression in the presence of doxorubicin (Figure 9C), indicating that imatinib-mediated inhibition of the HSP27/p38/Akt pathway involves inhibition of STAT3. Moreover, expression of a constitutively active p110α catalytic subunit of PI3K (E545K), which activates Akt (Figure 9D, bottom right), partially prevented imatinib-dependent potentiation of doxorubicin-induced PARP cleavage (Figure 9D). Thus, this is the first demonstration that imatinib prevents activation of a novel STAT3/HSP27/p38/Akt pathway, and that a HSP27/p38 pathway is involved in activating Akt during doxorubicin resistance. In summary, imatinib reverses intrinsic doxorubicin resistance by preventing STAT3 phosphorylation, which inhibits a HSP27/p38/Akt survival pathway and promotes activation of an NF-κB-mediated pro-apoptotic pathway (Figure 10).

## Discussion

Here, we show that imatinib prevents intrinsic and acquired resistance to doxorubicin by: 1) inhibiting c-Abl/Arg activation; 2) promoting doxorubicin-mediated cell cycle arrest at G2/M; 3) inhibiting activation of a STAT3-dependent HSP27/p38/Akt survival pathway; 4) promoting NF-κB-mediated inhibition of anti-apoptotic protein expression in a STAT3-dependent manner; and 5) inhibiting upregulation of the drug transporter, ABCB1, and directly inhibiting ABCB1 function (acquired resistance). These data are novel and significant because the upstream mechanisms that govern NF-κB-mediated transcriptional repression have not previously been identified. Furthermore, this is the first demonstration that HSP27/p38/Akt promote doxorubicin resistance in melanoma cells, and we are the first to show that STAT3 is involved in activation of this pathway.

The role of NF-κB in doxorubicin-induced cell death is controversial as doxorubicin-mediated activation of NF-κB prevents cell death in some cell types, while in other cells, doxorubicin-mediated activation of NF-κB promotes apoptosis by repressing expression of anti-apoptotic genes [16]–[20]. In addition, the mechanism by which anthracyclines convert NF-κB into a repressor also is under debate. Barker and colleagues showed that doxorubicin induces p65 nuclear localization and DNA binding of a non-acetylated/non-phosphorylated form of p65, which inhibits NF-κB transcriptional activity in a histone deacetylase (HDAC)-independent manner [19]. In contrast, Perkins and colleagues demonstrated that anthracyclines induce phosphorylation/acetylation and nuclear translocation of p65 in mouse embryo fibroblasts, and p65 represses gene expression by recruiting HDACs to gene targets [16]. In addition, Yu and colleagues showed that p65 acetylation is required for its nuclear retention, which is inconsistent with data from Barker and colleagues who demonstrate that non-phosphorylated/non-acetylated p65 binds DNA, and thus, is in the nucleus [19], [53]. Here, we show that doxorubicin induces p65 phosphorylation and nuclear translocation, which is enhanced by imatinib treatment or silencing STAT3, and correlates with decreased NF-κB transcriptional activity and downregulation of NF-κB targets. Thus, STAT3 activation inhibits doxorubicin-mediated p65 nuclear localization, which is contrary to data obtained in untreated cancer cells indicating that STAT3 promotes p65 nuclear retention [53]. Thus, our data indicate that STAT3 likely has an opposite role in regulating p65 nuclear localization in response to stimuli that convert NF-κB into a repressor [53].

Our data are consistent with Perkins and colleagues who demonstrate that doxorubicin increases NF-κB phosphorylation/acetylation/DNA binding but this activated NF-κB represses rather than activates transcription [16]. We provide additional data aimed at elucidating the mechanism by demonstrating that imatinib targets (likely c-Abl/Arg) and STAT3 regulate NF-κB function following doxorubicin treatment. It is possible that c-Abl/Arg and STAT3 prevent conversion of NF-κB into a repressor by promoting recruitment of histone acetyltransferases (HATs) to p65/p50-DNA complexes whereas inhibition of c-Abl/Arg or STAT3 promotes recruitment of histone deacetylases (HDACs) [16]. Interestingly, in cells that have acquired high-level resistance, doxorubicin increases NF-κB transcriptional activity, and NF-κB does not appear to function as a repressor. This may be because doxorubicin only modestly inhibits STAT3 phosphorylation, and thus, HAT recruitment may be dominant over HDAC recruitment in these cells. Addition of imatinib dramatically inhibits STAT3 and leads to repression of NF-κB targets (cIAP1, XIAP), perhaps by facilitating HDAC/p65 rather than HAT/p65 complexes. In either event, our data are highly significant because they demonstrate that imatinib converts a master survival regulator, NF-κB, from a pro-survival into a pro-apoptotic factor, thereby rendering a conventional chemotherapeutic agent more effective for treating metastatic disease. These data are extremely important because they indicate that NF-κB inhibitors may be ineffective in sensitizing tumors containing activated c-Abl/Arg to anthracyclines, and instead may antagonize anthracycline-induced apoptosis.

In addition to inhibiting survival signaling, we also demonstrate that c-Abl/Arg inhibitors partially prevent ABCB1 upregulation in cells that acquire doxorubicin resistance, and also directly inhibit ABCB1 function. c-Abl/Arg inhibitors have been identified as substrates of drug transporters in other cell types [7], [55]; however, this is the first demonstration that they inhibit ABCB1 in melanoma cells. In addition, we are the first to show that c-Abl promotes expression of an ABC transporter. ABC transporter upregulation has been shown to occur via a variety of pathways including FOXO3a and PI3K/Akt, NF-κB, HSP27, and ERK pathways [8]–[10]; ongoing experiments are aimed at identifying the mechanism of ABCB1 upregulation. ABCB1 also is a transporter for other chemotherapeutic agents, such as paclitaxel, vinblastine, vincristine, etoposide as well as for the selective estrogen receptor modulator (SERM), tamoxifen [7]. Thus, c-Abl/Arg inhibitors are likely to reverse resistance to many of these agents as well. In support of this hypothesis, we demonstrate that c-Abl/Arg inhibitors sensitize melanoma cells to paclitaxel, and Wang and colleagues showed that c-Abl/Arg inhibition sensitizes breast cancer cells to tamoxifen [56].

Doxorubicin is utilized to treat many cancers including triple-negative breast cancer; however, resistance and toxicity limit its effectiveness [2], [5]. Anthracyclines are not routinely used to treat metastatic melanoma due to intrinsic resistance; however, current treatment regimens (dacarbazine-DTIC or temozolamide-TMZ) are also ineffective (<5% success rate), and newer biological agents only extend survival by 4 months [57]–[59]. Our data demonstrate that c-Abl/Arg inhibitors (imatinib, nilotinib) are effective in reversing intrinsic and acquired doxorubicin resistance, thereby increasing doxorubicin effectiveness at lower doses. These data have important clinical ramifications as they indicate that Abl inhibitor/doxorubicin combinations may be effective for treating cancers driven by activated c-Abl and Arg.

## Supporting Information

Figure S1 **Genetic analysis of the BT-549 cell line.** DNA profile STR testing (DNA Safe) was performed by Genetic Testing Laboratory Inc. (GTL; Los Cruces, NM). The analysis revealed complete identity at all loci with the BT-549 breast cancer cell line (ATCC; Manassas, Va).
(PDF)

Figure S2 **c-Abl/Arg inhibitors reverses intrinsic doxorubicin resistance.** (**A**) WM3248 melanoma cells and (**B**) MDA-MB-468 breast cancer cells were treated with doxorubicin/imatinib (72 h) and viability assessed. Mean±SEM for 3 independent experiments (left) and representative dose response curves (right). (**C**) Graphical representation of combination indices obtained with CalcuSyn software (>1-antagonism;  = 1-additive; <1-synergism). (**D**) Parental (435s/M14) cells were treated with nilotinib/doxorubicin (72 h), and viability assessed. Mean±SEM for 3 independent experiments (left). Dose response curve is a representative experiment (right). (**E**) 293T cells expressing imatinib-resistant c-Abl (T) and Arg (T) were treated with imatinib (72 h) and blotted with antibodies. For all subfigures, IC_50_s represent Mean±SEM for 3 independent experiments; some error bars are too small to visualize. *p<0.05, ***p<0.001, using t-tests (see methods).
(PDF)

Figure S3 **c-Abl/Arg inhibition reverses acquired doxorubicin resistance.** (**A**) Parental 435s/M14 cells and their counterpart that acquired high-level doxorubicin resistance (DR) were serum-starved, and kinase activity assessed by *in vitro* kinase assay utilizing GST-Crk as substrate, and lysates blotted with the indicated antibodies. (**B**) Mean±SEM for 3 independent experiments for data shown in Fig. 1E. 435s/M14-DR - Dox (0.5 mM)+imatinib (10 mM), CI = 0.5; Dox (2 mM)+imatinib (10 mM), CI = 0.08. (**C**) Viability was assessed in nilotinib/doxorubicin-treated 435s/M14-DR cells. Mean±SEM for 3 independent experiments (left). Representative dose-response curve (right). For all subfigures, IC_50_s represent Mean±SEM for 3 independent experiments. *p<0.05, ***p<0.001, using t-tests (see methods).
(PDF)

Figure S4 **c-Abl/Arg inhibition reverses doxorubicin resistance by inhibiting proliferation and inducing apoptosis.** (**A**) MDA-MB-468 breast cancer and (**B**) WM-3248 melanoma cells were treated with doxorubicin and/or imatinib (72 h), and proliferation assessed by tritiated thymidine assay. Graphs shown are representative experiments. (A) MDA-MB-468 - Dox (0.005 mM)+imatinib (10 mM), CI = 1.1; Dox (0.01 mM)+imatinib (10 mM), CI = 0.9; (B) WM3248 - Dox (0.005 mM)+imatinib (10 mM), CI = 0.72; Dox (0.03 mM)+imatinib (10 mM), CI = 0.56. (**C**) Graphical representation of combination indices obtained with CalcuSyn software for data described in Fig. 2A,B in 435s/M14 (left) and BT-549 (right) cells. Graphs are representative of 3 independent experiments (>1-antagonism;  = 1-additive; <1-synergism). (**D**) 435s/M14-DR cells were treated with doxorubicin/imatinib (72 h), and proliferation assessed by tritiated thymidine assay. Representative dose response curve for data described in Fig. 2C. (**E**) Graphical representation of cells in G2/M phase for data shown in Fig. 2D,E. Mean±SEM from 3 independent experiments. (**F,G**) Cells were treated with doxorubicin/imatinib (40 h), and lysate from attached and detached cells was assessed for caspase-3/7 activity (**F**) or PARP cleavage (**G**). Representative experiments (from 3 independent experiments) are shown. For all figure parts: some error bars are too small for visualization. *p<0.05, ***p<0.001 using t-tests (see methods).
(PDF)

Figure S5 **Imatinib inhibits proliferation in the presence of doxorubicin via STAT3-dependent and independent mechanisms.** (**A–C**) 435s/M14 cells stably expressing pcDNA or STAT3C cells were treated with doxorubicin/imatinib (72 h), and analyzed by CellTiter-Glo viability assay (**A**), tritiated thymidine assay (**B**), or BrdU/PI FACS analysis (**C**). Representative experiments are shown on the left and Mean±SEM for three independent experiments are shown on the right (A,B,C). In some cases, error bars are too small to visualize. **p≤0.01 using t-tests (see methods).
(PDF)

Figure S6 **Imatinib potentiates doxorubicin-mediated inhibition of NF-kB targets.** (**A, B**) 435s/M14 cells were treated with imatinib (40 h) (**A**) or transfected with c-Abl/Arg siRNAs (**B**), and nuclear fractions analyzed by Western blot. Graphs are Mean±SEM from 3 independent experiments. WCL = whole cell lysate. (**C**) 435s/M14 cells stably expressing a 3X-NF-kB-luciferase reporter were treated with vehicle/imatinib (8 h) in the absence or presence of TNFa, and luciferase activity assessed. Mean±SEM for 3 independent experiments. (**D,E**) Graphical representation of Western blots (left) for data shown in Fig. 7C,D. Mean±SEM for 3 independent experiments. (**F**) A second graphical representation (Mean±SEM for 3 independent experiments) for PARP cleavage data presented in Fig. 7E, left, which demonstrates that the rescue of PARP cleavage following silencing p65 is not complete. For all subfigures: some error bars are too small to visualize. *p<0.05, **p≤0.01, ***p<0.001 (see methods).
(PDF)

Figure S7 **Imatinib inhibits induction of a STAT3-dependent HSP27/p38/Akt survival pathway in response to doxorubicin treatment.** (**A**) Graphical representation of Western blots described in Fig. 9A. Mean±SEM for 3 independent experiments. (**B**) 435s/M14-DR cells were treated with imatinib and/or doxorubicin (40 h), and lysates from attached and detached cells were analyzed by Western blot. Graph represents Mean±SEM for 3 independent experiments. (**C**) 435s/M14 cells, transfected with STAT3 siRNA, were treated with doxorubicin (40h), and lysates blotted with antibodies. Graphs represent Mean±SEM from 3 independent experiments for data described in Fig. 9B. For all subfigures: some error bars are too small to visualize. *p<0.05, **p≤0.01, ***p<0.001 (see methods).
(PDF)
