# Supplementary material for: Imatinib Reverses Doxorubicin Resistance by Affecting Activation of STAT3-Dependent NF-κB and HSP27/p38/AKT Pathways and by Inhibiting ABCB1
Source: PLoS One. 2013 Jan 31;8(1):e55509. doi: 10.1371/journal.pone.0055509 (PMC3561297; doi:10.1371/journal.pone.0055509)
Supplement: Figure S1 — Genetic analysis of the BT-549 cell line. DNA profile STR testing (DNA Safe) was performed by Genetic Testing Laboratory Inc. (GTL; Los Cruces, NM). The analysis revealed complete identity at all loci with the BT-549 breast cancer cell line (ATCC; Manassas, Va). (PDF) [file pone.0055509.s001.pdf]

Genetic Testing Laboratories
MSC3ARP, Box 30001
Las Cruces, New Mexico
(866) 833-6895

AABB/ISO Accredited Testing Facility

DNA Safe Autosomal DNA Analysis

Photo ID's for the persons submitting these samples were not verified by witness.

<*ORIGINAL SIGNED REPORT IN COLOR*>

Client Identification: Rina Plattner	OID: L11-6674	Report Release Date: 06/17/2011 NLB ANA AUTO	Page 1 of 1

DNA Donor's Name: BT-549 Cell Line			
Date Collected: Unknown			
**STR Locus**	**Alleles Called**		
D3S1358		18	
TH01		9.3	
D21S11		32.2	
D18S51		15	
Penta E		14	
D5S818		11	
D13S317	11		12
D7S820	9		10
D16S539		8	
CSF1PO	10		12
Penta D		13	
**Amelogenin**	Female (XX)		
vWA		15	
D8S1179		16	
TPOX		8	
FGA		19	

Laboratory Batch Number:	111673130C1

Understanding This Analysis

DNASafe™, an individual DNA profile, is a permanent means of individual identification. Unlike a name that may be shared, a social security number that can be stolen, or photographs that change over time, your personal DNA identity remains constant from the moment of conception to the end of life. Your DNASafe™ profile demonstrates your genetic similarity to family members as well as the genetic uniqueness that distinguishes you from the rest of the world.

The Genetic Testing Laboratories, Inc. is AABB and ISO 17025:2005 Accredited. These accreditations set the global standard for the technical competence of DNA testing laboratories. You may have complete confidence in these results.

Comments:

For legal and criminal casework, Chain of Custody documentation is included with this report. DNA amplified with PowerPlex16® System, detected using the ABI PRISM® 3130xl Genetic Analyzer and analyzed with GeneMapper® ID Software. Testing performed by GTL Incorporated, a division of General Genetics Corporation. GTL is an AABB, A2LA, NATA and ISO 17025-2005 Accredited Facility.

_______________ / 06/17/2011
Dan J. Slowinski / Date (mm/dd/yy)
Laboratory Manager, GTL

R-010 REV-9 CHG-0.doc

Record Consists of 1 Page

BT-549 ATCC Reference Profile
NCI-60 Panel (Lorenzi et al. 2009)

STR LOCUS	Alleles
D3S1358	N.D.
TH01	9.3
D21S11	N.D.
Penta E	N.D.
D5S818	11
D13S317	N.D.
D7S820	9,10
D16S539	8
CSF1PO	10,12
Penta D	N.D.
Amelogenin	X
vWA	15
D8S1179	N.D.
TPOX	8
FGA	N.D.
