# Supplementary figures and images for: Imatinib Reverses Doxorubicin Resistance by Affecting Activation of STAT3-Dependent NF-κB and HSP27/p38/AKT Pathways and by Inhibiting ABCB1
Source: PLoS One. 2013 Jan 31;8(1):e55509. doi: 10.1371/journal.pone.0055509 (PMC3561297; doi:10.1371/journal.pone.0055509)

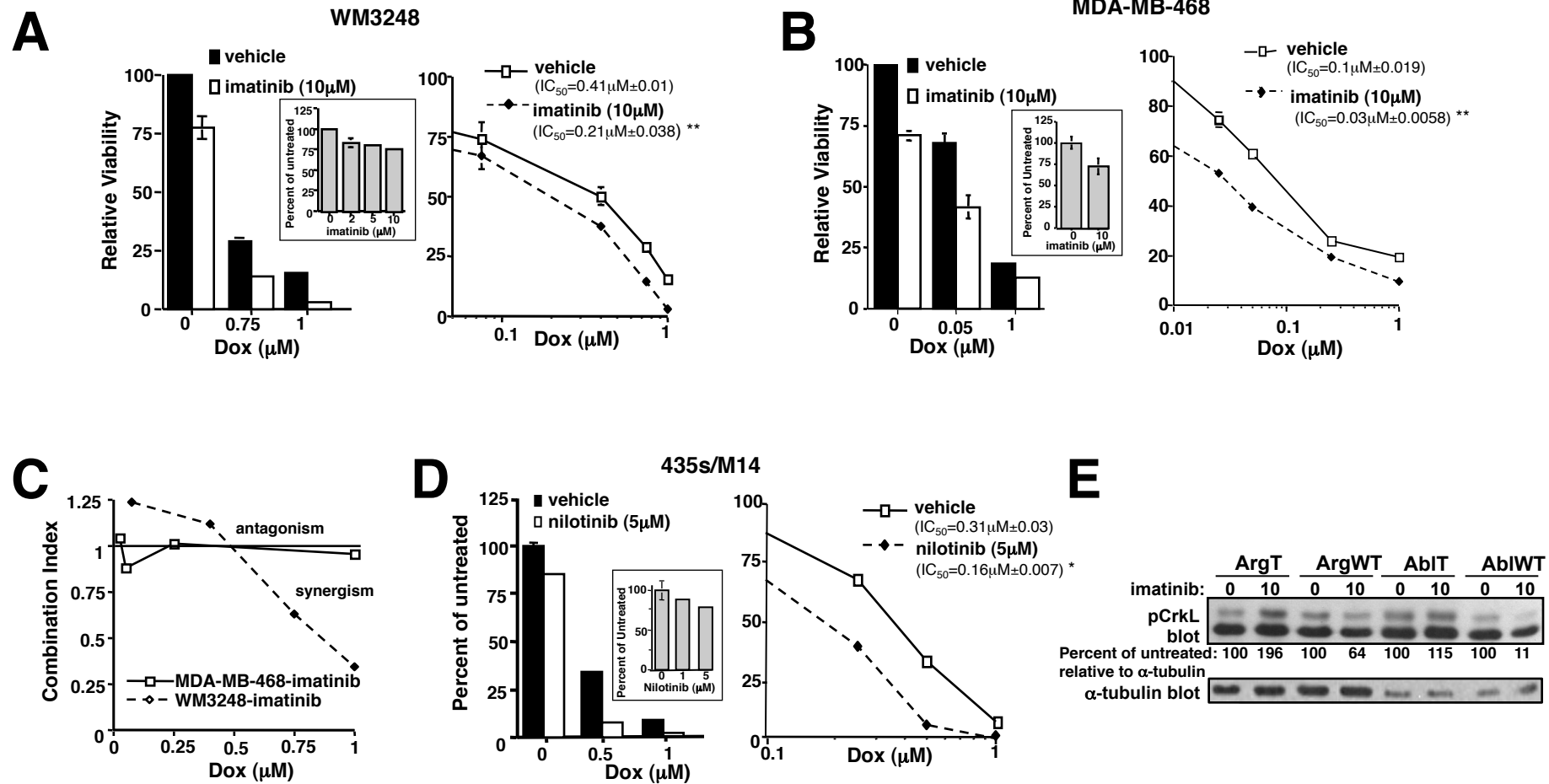

Supplement: Figure S2 — c-Abl/Arg inhibitors reverses intrinsic doxorubicin resistance. (A) WM3248 melanoma cells and (B) MDA-MB-468 breast cancer cells were treated with doxorubicin/imatinib (72 h) and viability assessed. Mean±SEM for 3 independent experiments (left) and representative dose response curves (right). (C) Graphical representation of combination indices obtained with CalcuSyn software (>1-antagonism; = 1-additive; <1-synergism). (D) Parental (435s/M14) cells were treated with nilotinib/doxorubicin (72 h), and viability assessed. Mean±SEM for 3 independent experiments (left). Dose response curve is a representative experiment (right). (E) 293T cells expressing imatinib-resistant c-Abl (T) and Arg (T) were treated with imatinib (72 h) and blotted with antibodies. For all subfigures, IC50s represent Mean±SEM for 3 independent experiments; some error bars are too small to visualize. *p<0.05, ***p<0.001, using t-tests (see methods). (PDF) [file pone.0055509.s002.pdf]

**A**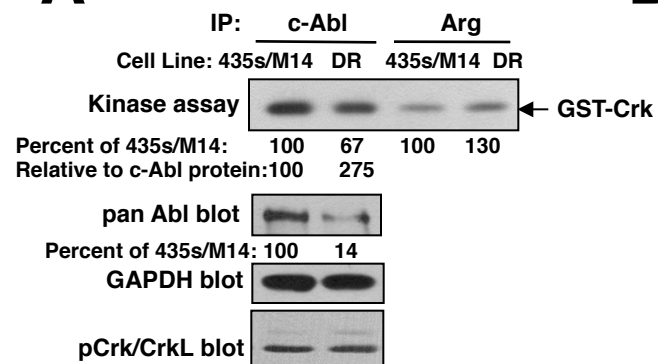**B**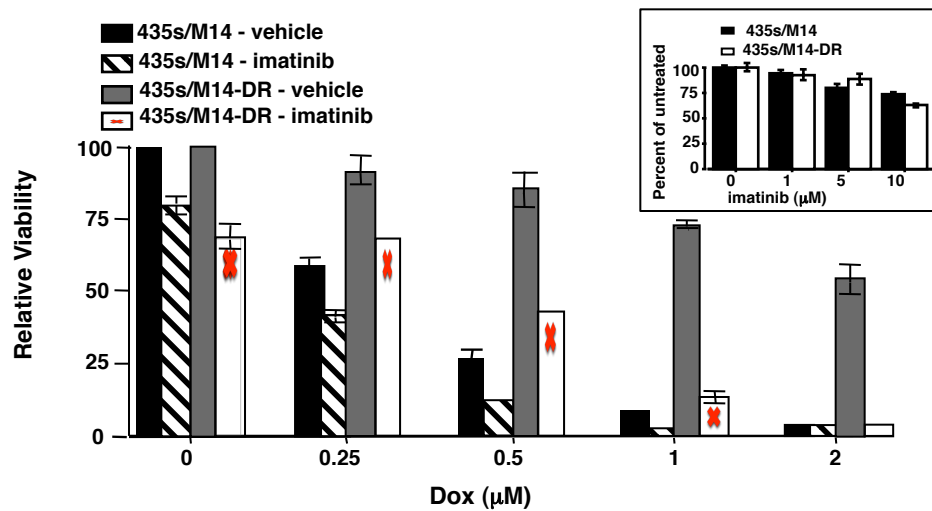**C**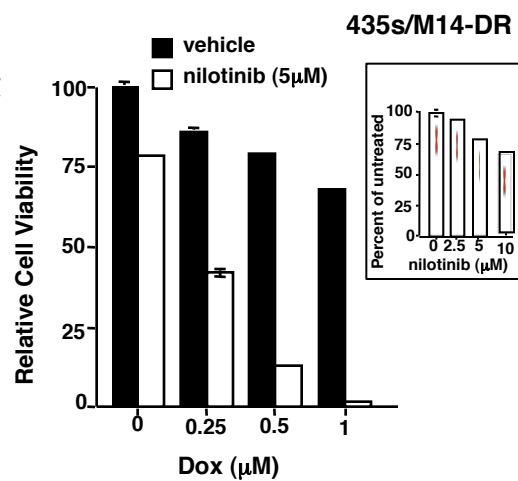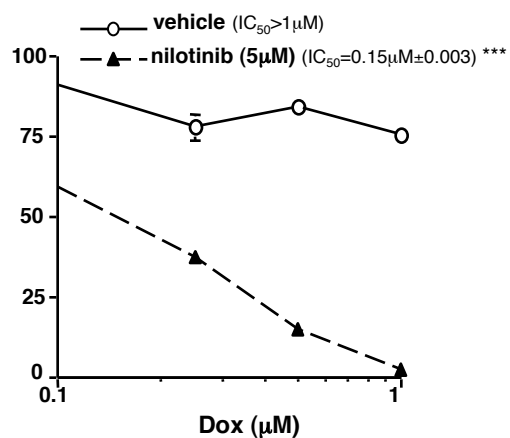

Supplement: Figure S3 — c-Abl/Arg inhibition reverses acquired doxorubicin resistance. (A) Parental 435s/M14 cells and their counterpart that acquired high-level doxorubicin resistance (DR) were serum-starved, and kinase activity assessed by in vitro kinase assay utilizing GST-Crk as substrate, and lysates blotted with the indicated antibodies. (B) Mean±SEM for 3 independent experiments for data shown in Fig. 1E. 435s/M14-DR - Dox (0.5 mM)+imatinib (10 mM), CI = 0.5; Dox (2 mM)+imatinib (10 mM), CI = 0.08. (C) Viability was assessed in nilotinib/doxorubicin-treated 435s/M14-DR cells. Mean±SEM for 3 independent experiments (left). Representative dose-response curve (right). For all subfigures, IC50s represent Mean±SEM for 3 independent experiments. *p<0.05, ***p<0.001, using t-tests (see methods). (PDF) [file pone.0055509.s003.pdf]

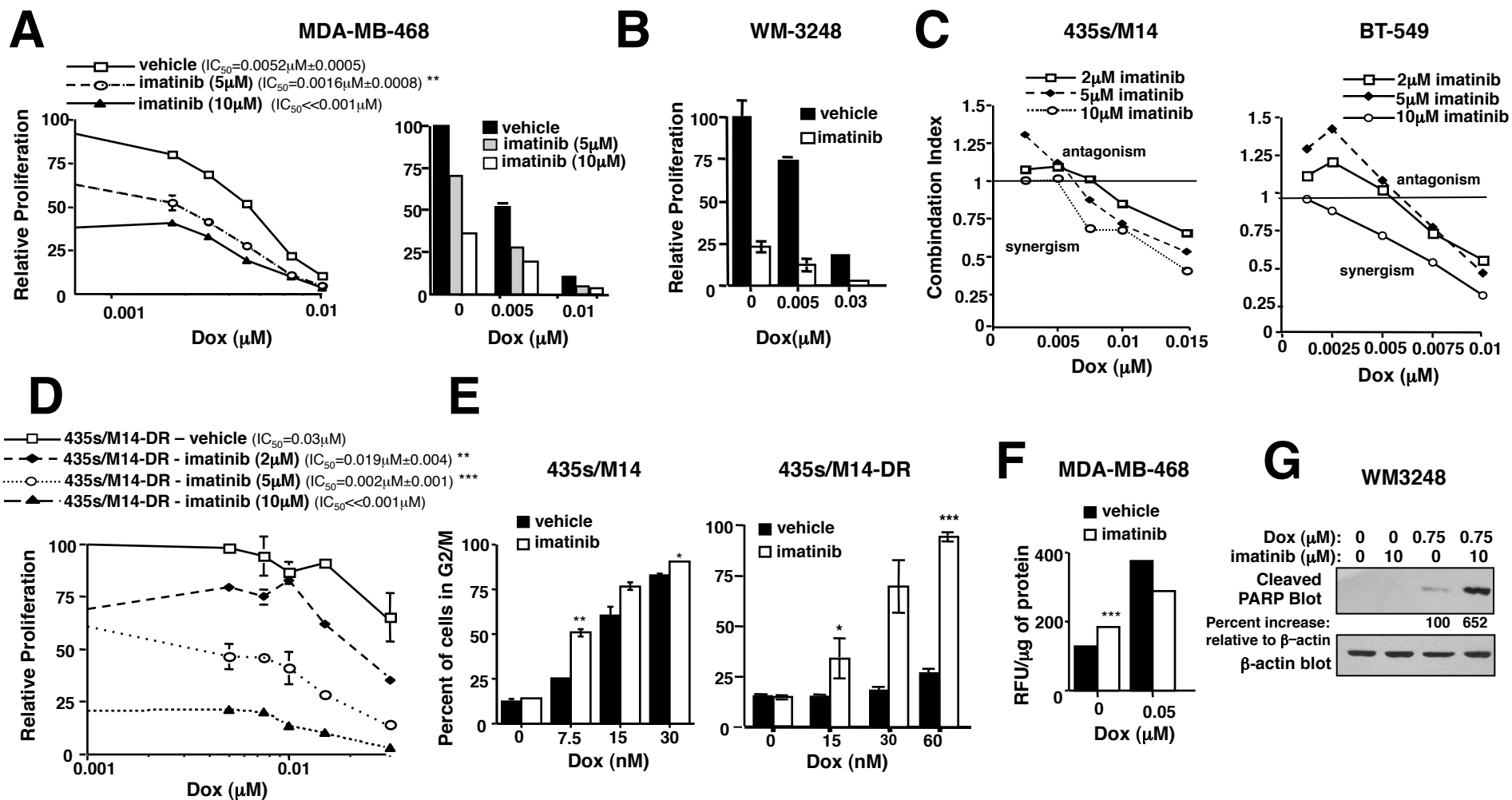

Supplement: Figure S4 — c-Abl/Arg inhibition reverses doxorubicin resistance by inhibiting proliferation and inducing apoptosis. (A) MDA-MB-468 breast cancer and (B) WM-3248 melanoma cells were treated with doxorubicin and/or imatinib (72 h), and proliferation assessed by tritiated thymidine assay. Graphs shown are representative experiments. (A) MDA-MB-468 - Dox (0.005 mM)+imatinib (10 mM), CI = 1.1; Dox (0.01 mM)+imatinib (10 mM), CI = 0.9; (B) WM3248 - Dox (0.005 mM)+imatinib (10 mM), CI = 0.72; Dox (0.03 mM)+imatinib (10 mM), CI = 0.56. (C) Graphical representation of combination indices obtained with CalcuSyn software for data described in Fig. 2A,B in 435s/M14 (left) and BT-549 (right) cells. Graphs are representative of 3 independent experiments (>1-antagonism; = 1-additive; <1-synergism). (D) 435s/M14-DR cells were treated with doxorubicin/imatinib (72 h), and proliferation assessed by tritiated thymidine assay. Representative dose response curve for data described in Fig. 2C. (E) Graphical representation of cells in G2/M phase for data shown in Fig. 2D,E. Mean±SEM from 3 independent experiments. (F,G) Cells were treated with doxorubicin/imatinib (40 h), and lysate from attached and detached cells was assessed for caspase-3/7 activity (F) or PARP cleavage (G). Representative experiments (from 3 independent experiments) are shown. For all figure parts: some error bars are too small for visualization. *p<0.05, ***p<0.001 using t-tests (see methods). (PDF) [file pone.0055509.s004.pdf]

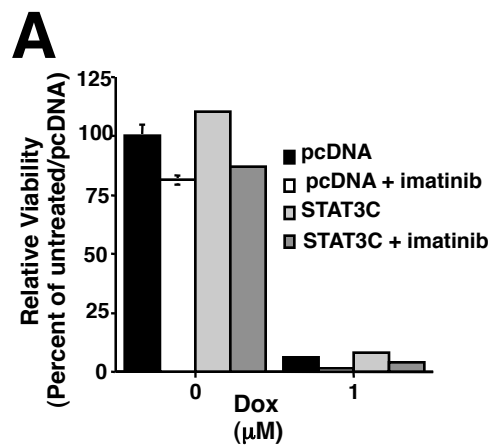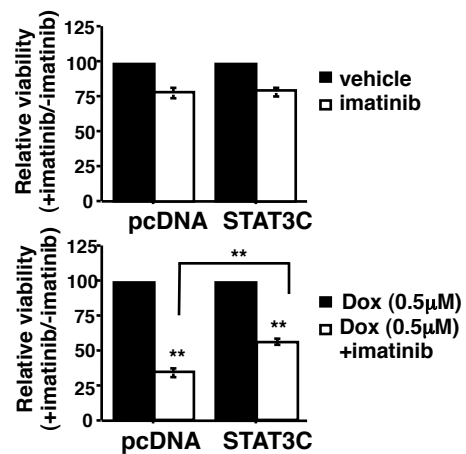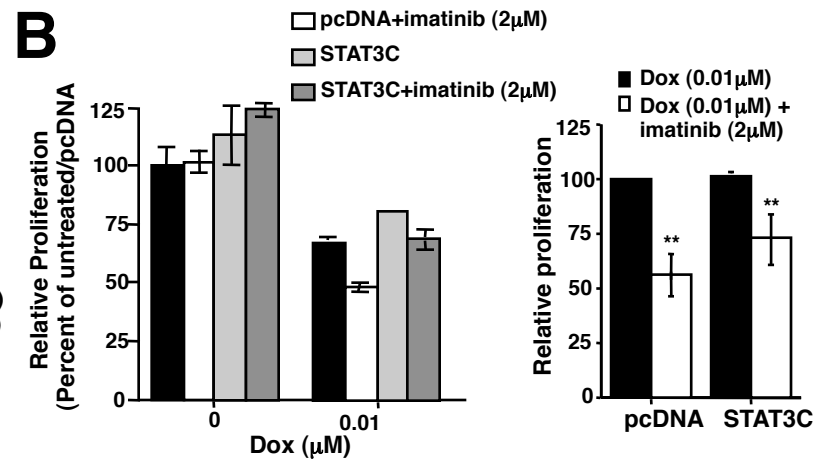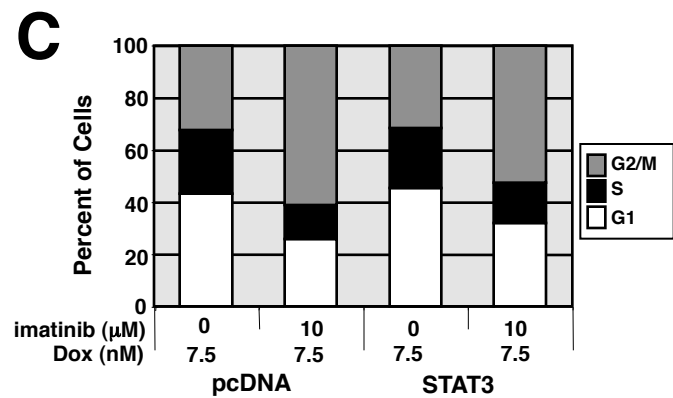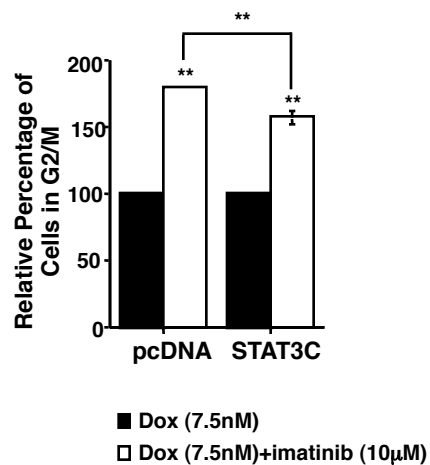

Supplement: Figure S5 — Imatinib inhibits proliferation in the presence of doxorubicin via STAT3-dependent and independent mechanisms. (A–C) 435s/M14 cells stably expressing pcDNA or STAT3C cells were treated with doxorubicin/imatinib (72 h), and analyzed by CellTiter-Glo viability assay (A), tritiated thymidine assay (B), or BrdU/PI FACS analysis (C). Representative experiments are shown on the left and Mean±SEM for three independent experiments are shown on the right (A,B,C). In some cases, error bars are too small to visualize. **p≤0.01 using t-tests (see methods). (PDF) [file pone.0055509.s005.pdf]

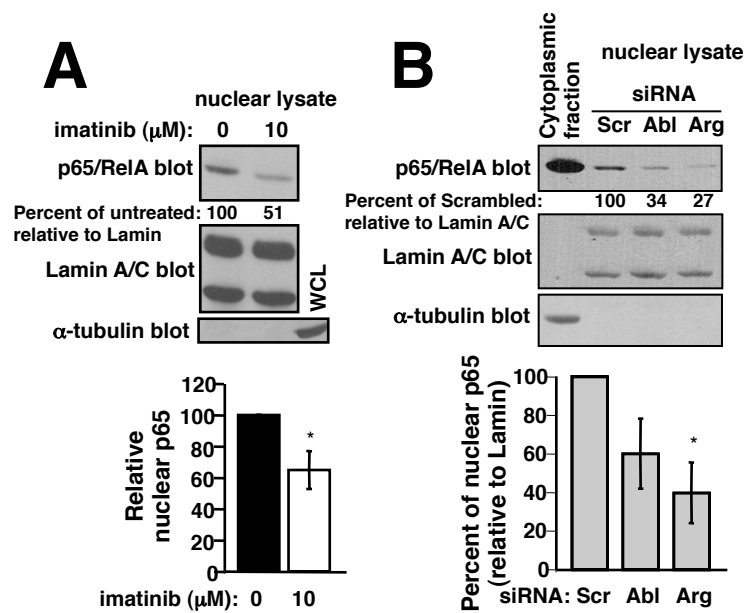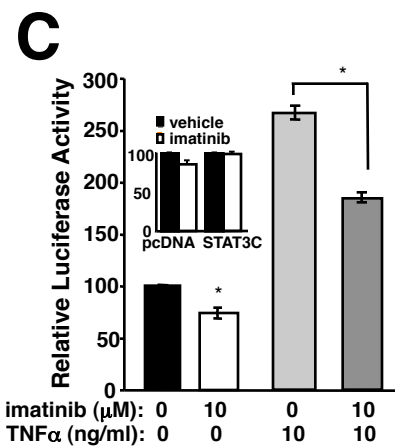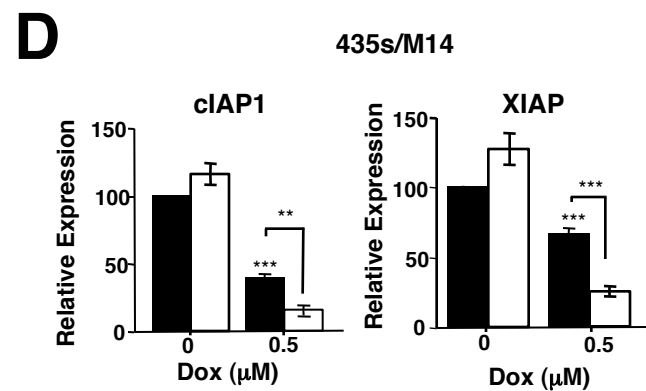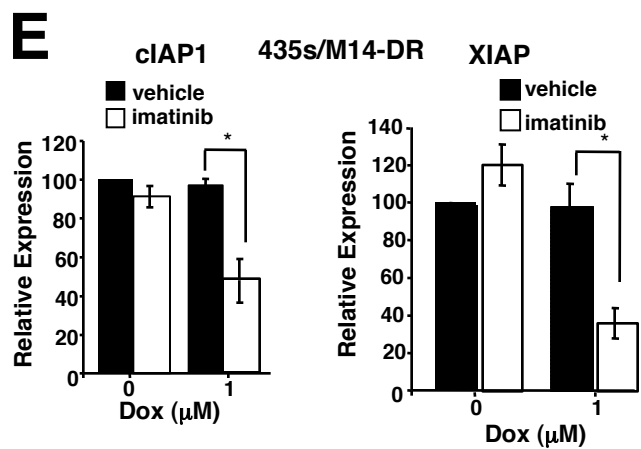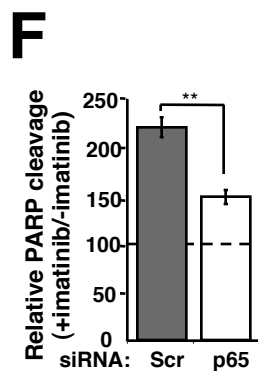

Supplement: Figure S6 — Imatinib potentiates doxorubicin-mediated inhibition of NF-kB targets. (A, B) 435s/M14 cells were treated with imatinib (40 h) (A) or transfected with c-Abl/Arg siRNAs (B), and nuclear fractions analyzed by Western blot. Graphs are Mean±SEM from 3 independent experiments. WCL = whole cell lysate. (C) 435s/M14 cells stably expressing a 3X-NF-kB-luciferase reporter were treated with vehicle/imatinib (8 h) in the absence or presence of TNFa, and luciferase activity assessed. Mean±SEM for 3 independent experiments. (D,E) Graphical representation of Western blots (left) for data shown in Fig. 7C,D. Mean±SEM for 3 independent experiments. (F) A second graphical representation (Mean±SEM for 3 independent experiments) for PARP cleavage data presented in Fig. 7E, left, which demonstrates that the rescue of PARP cleavage following silencing p65 is not complete. For all subfigures: some error bars are too small to visualize. *p<0.05, **p≤0.01, ***p<0.001 (see methods). (PDF) [file pone.0055509.s006.pdf]

**A**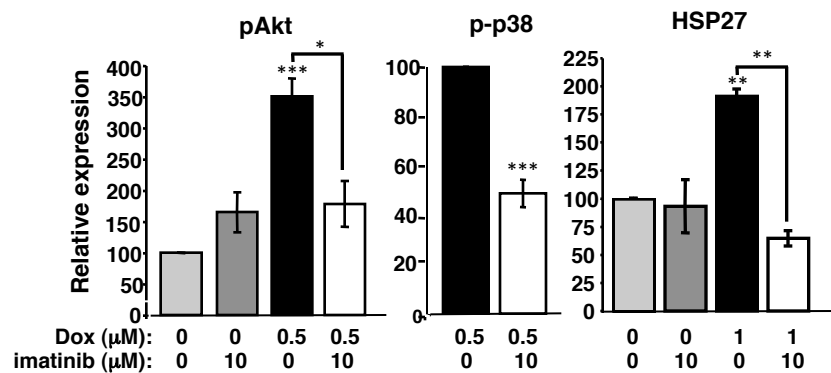**B**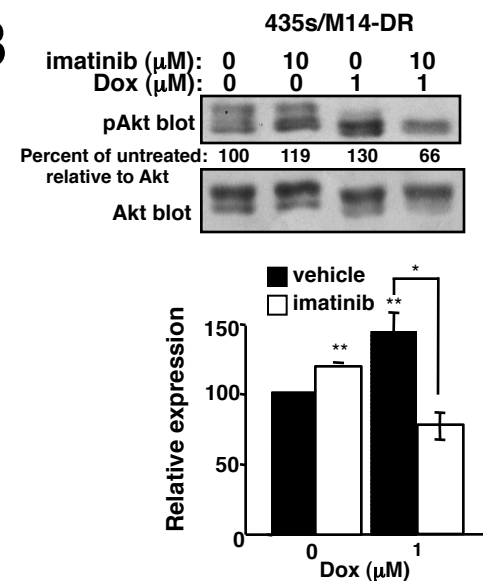**C**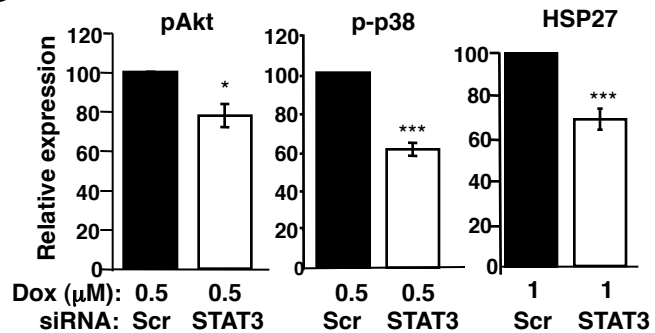

Supplement: Figure S7 — Imatinib inhibits induction of a STAT3-dependent HSP27/p38/Akt survival pathway in response to doxorubicin treatment. (A) Graphical representation of Western blots described in Fig. 9A. Mean±SEM for 3 independent experiments. (B) 435s/M14-DR cells were treated with imatinib and/or doxorubicin (40 h), and lysates from attached and detached cells were analyzed by Western blot. Graph represents Mean±SEM for 3 independent experiments. (C) 435s/M14 cells, transfected with STAT3 siRNA, were treated with doxorubicin (40h), and lysates blotted with antibodies. Graphs represent Mean±SEM from 3 independent experiments for data described in Fig. 9B. For all subfigures: some error bars are too small to visualize. *p<0.05, **p≤0.01, ***p<0.001 (see methods). (PDF) [file pone.0055509.s007.pdf]
